# A Compartmentalized Reduction in Membrane-Proximal Calmodulin Reduces the Immune Surveillance Capabilities of CD8^+^ T Cells in Head and Neck Cancer

**DOI:** 10.3389/fphar.2020.00143

**Published:** 2020-02-28

**Authors:** Ameet A. Chimote, Vaibhavkumar S. Gawali, Hannah S. Newton, Trisha M. Wise-Draper, Laura Conforti

**Affiliations:** ^1^ Division of Nephrology, Department of Internal Medicine, University of Cincinnati, Cincinnati, OH, United States; ^2^ Division of Hematology Oncology, Department of Internal Medicine, University of Cincinnati, Cincinnati, OH, United States

**Keywords:** ion channels, T cells, head and neck cancer, KCa3.1, calmodulin, T cell function, T cell chemotaxis

## Abstract

The limited ability of cytotoxic CD8^+^ T cells to infiltrate solid tumors and function within the tumor microenvironment presents a major roadblock to effective immunotherapy. Ion channels and Ca^2+^-dependent signaling events control the activity of T cells and are implicated in the failure of immune surveillance in cancer. Reduced KCa3.1 channel activity mediates the heightened inhibitory effect of adenosine on the chemotaxis of circulating T cells from head and neck squamous cell carcinoma (HNSCC) patients. Herein, we conducted experiments that elucidate the mechanisms of KCa3.1 dysfunction and impaired chemotaxis in HNSCC CD8^+^ T cells. The Ca^2+^ sensor calmodulin (CaM) controls multiple cellular functions including KCa3.1 activation. Our data showed that CaM expression is lower in HNSCC than healthy donor (HD) T cells. This reduction was due to an intrinsic decrease in the genes encoding CaM combined to the failure of HNSCC T cells to upregulate CaM upon activation. Furthermore, the reduction in CaM was confined to the plasma membrane and resulted in decreased CaM-KCa3.1 association and KCa3.1 activity (which was rescued by the delivery of CaM). IFNγ production, also Ca^2+^- and CaM-dependent, was instead not reduced in HNSCC T cells, which maintained intact cytoplasmic CaM and Ca^2+^ fluxing ability. Knockdown of CaM in HD T cells decreased KCa3.1 activity, but not IFNγ production, and reduced their chemotaxis in the presence of adenosine, thus recapitulating HNSCC T cell dysfunction. Activation of KCa3.1 with 1-EBIO restored the ability of CaM knockdown HD T cells to chemotax in the presence of adenosine. Additionally, 1-EBIO enhanced INFγ production. Our data showed a localized downregulation of membrane-proximal CaM that suppressed KCa3.1 activity in HNSCC circulating T cells and limited their ability to infiltrate adenosine-rich tumor-like microenvironments. Furthermore, they indicate that KCa3.1 activators could be used as positive CD8^+^ T cell modulators in cancers.

## Introduction

The immune system is the first line of defense against cancer. Indeed, in several solid malignancies, including head and neck squamous cell carcinomas (HNSCC), an increased infiltration of cytotoxic CD8^+^ T cells is associated with a favorable patient prognosis and therapeutic response (Balermpas et al., 2014; Fang et al., 2017). However, the ability of CD8^+^ T cells to infiltrate the tumor microenvironment (TME) and produce an anti-tumor response is often severely compromised (Uppaluri et al., 2008; Joyce and Fearon, 2015; Gurusamy et al., 2017). Ion channels have been implicated in this failure of immune surveillance in cancer (Chimote et al., 2017; Chimote et al., 2018).

Ion channels regulate multiple T lymphocyte functions including cytokine production, cytotoxicity, and motility, which are vital for generating an anti-tumor immune response (Cahalan and Chandy, 2009; Feske et al., 2012; Kuras et al., 2012; Zhong et al., 2013; Chimote et al., 2018). In particular, two K^+^ channels, the voltage-dependent Kv1.3 and the Ca^2+^-activated KCa3.1, regulate the electrochemical driving force for Ca^2+^ influx that is necessary for T cell effector functions (Cahalan and Chandy, 2009; Feske et al., 2012). Furthermore, KCa3.1 channels localize at the uropod of migrating human T cells and control chemotaxis (Kuras et al., 2012). Blockade of these channels suppresses T cell effector functions, motility, and chemotaxis (Cahalan and Chandy, 2009; Kuras et al., 2012). Indeed, Kv1.3 and KCa3.1 activity is reduced in tumor infiltrating lymphocytes (TIL) and circulating T cells of HNSCC patients, respectively, thus contributing to the failure of immune surveillance (Chimote et al., 2017; Chimote et al., 2018). Ultimately, restoring these channels' activity ameliorates T cell functionality, and overexpression of Kv1.3 decreases tumor burden and increases survival in tumor bearing mice (Eil et al., 2016; Chimote et al., 2018). Thus, understanding the causes of the abnormal behavior of these channels in cancer patients could lead to the discovery of new therapeutic targets for improved immune therapies in cancer.

Kv1.3 and KCa3.1 channels are suppressed by elements of the TME including hypoxia and adenosine (Chimote et al., 2012; Chimote et al., 2013; Chimote et al., 2017; Chimote et al., 2018). KCa3.1 channels, in particular, provide a link between adenosine and reduced T cell effector functions (Chimote et al., 2013). Adenosine, which is increased in TME of solid tumors, acts on T cells through the A_2A_ adenosine receptor (A_2A_R), which in turn activates cAMP and PKA and, ultimately, inhibits KCa3.1, thus suppressing T cell motility and cytokine release (Chimote et al., 2013; Chimote et al., 2018). This immune suppressive pathway is particularly troublesome in HNSCC patients where CD8^+^ T cells have an increased sensitivity to adenosine than healthy donors (HD) (Mandapathil et al., 2012; Chimote et al., 2018). The latter heightened response to adenosine is due to the diminished KCa3.1 function in HNSCC CD8^+^ T cells, which is corrected by addition of KCa3.1 positive modulators (Chimote et al., 2018). Thus, defective KCa3.1 channel function can provide an explanation for the decreased immune surveillance in the adenosine-rich TME (Chimote et al., 2018). However, the mechanisms mediating these abnormalities in CD8^+^ T cells of HNSCC patients are not known.

Calmodulin (CaM) gene expression has been shown to be altered in TIL of breast cancer patients (Gu-Trantien et al., 2013). CaM is a signaling molecule that controls multiple T cell functions including KCa3.1 activity which is triggered by an increase in cytosolic Ca^2+^ (Cahalan and Chandy, 2009; Sforna et al., 2018). CaM binds to the C-terminus of KCa3.1 and acts as a calcium sensor (Schumacher et al., 2001; Stocker, 2004; Adelman, 2016; Lee and Mackinnon, 2018; Sforna et al., 2018). CaM is constitutively bound to the channel in a Ca^2+^-independent manner (Schumacher et al., 2001; Stocker, 2004; Adelman, 2016; Lee and Mackinnon, 2018). This pre-association allows for rapid channel gating when the intracellular Ca^2+^ concentration changes (Adelman, 2016). When intracellular Ca^2+^ increases, CaM undergoes conformational changes and causes the channel to open (Schumacher et al., 2001; Stocker, 2004; Lee and Mackinnon, 2018; Sforna et al., 2018). While it is known that CaM levels increase in activated healthy T cells, its protein abundance and functional implications in T cells of cancer patients, to our knowledge, have not yet been investigated (Colomer et al., 1993). In this study, we provide evidence of a compartmentalized reduction of CaM levels at the plasma membrane of CD8^+^ T cells of HNSCC patients that selectively impacts KCa3.1 activity and chemotaxis. This mechanism may ultimately compromise immune surveillance in cancer.

## Materials and Methods

### Human Subjects

Peripheral blood was obtained from 19 de-identified HNSCC patients in the age range of 47 to 84. The eligibility criteria for patient inclusion in the study were a positive diagnosis for HNSCC confirmed by tissue biopsy and no administration of radiation or chemotherapy before the time of drawing the blood (see **Table 1** for a summary of patient demographics and **Table S1** for clinical information). The data on the study subjects were collected and managed using Research Electronic Data Capture (REDCap) tools hosted at the University of Cincinnati. Peripheral blood was also drawn from seven age-matched ( ± 5 years) HD (five female and two male) in the age range of 41 to 67 years. Informed consent was obtained from all HNSCC patients and HDs. The study and informed consent forms were approved by the University of Cincinnati Institutional Review Board (IRB no. 2014-4755). As healthy controls, we also utilized discarded blood units from Hoxworth Blood Center (University of Cincinnati) for our studies. Age information about these samples are not available.

**Table 1:** Demographics of HNSCC patients enrolled in the study.

Age (at the time of sample collection)	Years
Range	47–84
Mean	60
Variable	Number (%)
Gender	
Male	14 (74)
Female	5 (26)
Site	
Oral Cavity	6 (32)
Oropharynx	7 (37)
Larynx	5 (26)
Hypopharynx	1 (5)
Nasopharynx	0 (0)
Tumor stage	
T1	4 (21)
T2	8 (42)
T3	3 (16)
T4	4 (21)
Unknown	0 (0)
Nodal Status	
N0	6 (32)
N1	1 (5)
N2	10 (53)
N3	1 (5)
Unknown	1 (5)
ECOG (Eastern Cooperative Oncology Group) performance status	
0	8 (42)
1	4 (21)
2	2 (11)
Unknown	5 (26)
Smoking	
No (< 10 pk years)	9 (47)
Yes (> 10 pk years)	10 (53)
Alcohol	
No	16 (84)
Yes (> 5 drinks/week)	3 (16)
p16 status	
Positive	7 (37)
Negative	7 (37)
Unknown	5 (26)

HNSCC patients (n = 19) were enrolled in the study according to eligibility criteria. TNM classification was used to stage tumor size and nodal involvement. T1 to T4 refers to the size and invasion of the tumors. N1 to N3 refers to the number and location of the regional lymph nodes involved. Performance status was measured by ECOG score, a 0 to 5 scale that describes the daily living abilities of the patient. Smoking status was calculated by multiplying the number of packs of cigarettes smoked per day by the number of years the individual has smoked (pk years). A cut off of 10 pk years was used to differentiate smoking status.

### Reagents and Chemicals

Human serum, L-glutamine, adenosine, bovine brain calmodulin, 1-EBIO, sodium hydroxide, thapsigargin, poly-L-lysine, and Tween-20 were purchased from MilliporeSigma. Hepes, RPMI 1640, fetal bovine serum, penicillin, streptomycin, L-glutamine, Hepes, and phosphate-buffered saline (PBS) were obtained from Gibco (ThermoFisher). Collagen I, rat tail was obtained from Corning Inc. CXCL12 was obtained from R&D Systems. Stock solution of adenosine was prepared in sterile water. Stock solution of CXCL12 was prepared in sterile PBS containing 0.1% bovine serum albumin. Stock solutions of 1-EBIO and thapsigargin were prepared in dimethyl sulfoxide and used at 0.1% dilution.

### Cell Isolation and *In Vitro* Activation

Peripheral blood mononuclear cells (PBMCs) were isolated from whole blood by Ficoll-Paque density gradient centrifugation (GE Healthcare Bio-Sciences), as described previously (Chimote et al., 2018). CD8^+^ T cells were isolated from PBMCs by negative selection using the EasySep Human CD8^+^ T Cell Enrichment Kit (STEMCELL Technologies Inc.). The CD8^+^ T cells were maintained in RPMI 1640 medium supplemented with 10% human serum, penicillin (200 U/ml), streptomycin (200 μg/ml), 1 mM L-glutamine, and 10 mM Hepes. For all experiments, cells were activated in a cell culture dish pre-coated with mouse anti-human CD3 antibody (10 μg/ml) (BioLegend) and mouse anti-human CD28 antibody (10 μg/ml) (BioLegend) for 72 to 96 h, except where specified.

### Reverse Transcription Quantitative Polymerase Chain Reaction

Total RNA was isolated from resting and activated HD and HNSCC CD8^+^ T cells using the E.Z.N.A. total RNA isolation Kit (Omega Bio-tek). 423 ng of RNA was used to synthesize complementary DNA (cDNA) using the Maxima First Strand cDNA Synthesis Kit for RT-qPCR (ThermoFisher). Predesigned primers for RT-qPCR were obtained using TaqMan Gene Expression Assays (Applied Biosystems, Thermo Fisher) to detect the expression of *CALM1* (assay ID: Hs00300085_s1), *CALM2* (assay ID: Hs00830212_s1), *CALM3* (assay ID: Hs00968732_g1), *18s rRNA* (assay ID: Hs99999901_s1), and *GAPDH* (assay ID: HS03929097_g1). The RT-qPCR was set up in a 96-well plate by adding 30 ng of cDNA, 1× TaqMan Gene Expression Master Mix (Applied Biosystems, ThermoFisher), and 1 μl of TaqMan Gene Expression Assay primers. All samples were run in technical replicates of quadruplicates or triplicates, as indicated in the individual figure or table legends. *18s rRNA* was used as an internal control. RT-qPCR was cycled in Applied Biosystems StepOne Real-Time PCR System (Applied Biosystems). C_T_ values were measured using StepOne software version 2.1 (Applied Biosystems). C_T_ values for *CALM1*, *CALM2*, and *CALM3* were normalized against measured C_T_ values for *18s rRNA*, and the ΔΔC_T_ values were calculated as described previously (Chimote et al., 2018). Relative quantity (RQ) values, representing the fold change in *CALM1*, *CALM2*, and *CALM3* gene expression in resting as compared to activated HD and HNSCC CD8^+^ T cells, were calculated as the 2^−ΔΔC_T_^ values. To investigate the verity of *18s rRNA* as a housekeeping gene for our experiments, we measured *CALM1* expression in resting and activated CD8^+^ T cells from a single HNSCC patient using either *18s rRNA* or *GAPDH* as housekeeping genes (**Table S2**). The C_T_ values for *CALM1* were normalized against measured C_T_ values for *18s rRNA*, as well against C_T_ values for *GAPDH* in resting and activated HNSCC CD8^+^ T cells. We also normalized the C_T_ values for *GAPDH* against the C_T_ values of *18s rRNA*. RQ values were calculated as 2^−ΔΔC_T_^ values as described above and the results are presented in **Table S2**.

### Immunofluorescence and Confocal Microscopy

2 X 10^5^ activated CD8^+^ T cells from HDs and HNSCC patients were plated on poly-L-lysine-coated coverslips in a four-well tissue culture plate, fixed in 1% paraformaldehyde, and blocked with a solution of PBS containing 10% FBS for 1 h at room temperature. Cells were then stained overnight at 4°C with either Alexa 488-conjugated mouse anti-human CD8 antibody (Clone SPM548, Novus Biologicals) or biotin-conjugated mouse anti-human KCa3.1 antibody (Clone 6C1, Alomone labs) diluted in blocking solution. The background staining of the Biotin conjugated KCa3.1 antibody is shown in **Figure S1**. The following day, coverslips labeled with the biotin-conjugated primary were incubated with Alexa Fluor 488 conjugated-streptavidin secondary antibody (Biolegend) for 1 h at room temperature. All coverslips were then permeabilized with PBS + 0.2% Triton X-100 for 20 min at room temperature, blocked with blocking solution for 1 h, and stained with Alexa Fluor 647–conjugated mouse anti-human CaM antibody (clone 2D1, Novus Biologicals) diluted in blocking solution for 4 h at room temperature. Cells were washed thoroughly with PBS-T washing solution and coverslips were mounted on glass slides using Fluoromount G (eBiosciences, ThermoFisher). Cells were visualized by confocal microscopy (Zeiss LSM 710 laser scanning microscope, Zeiss GmbH) using a 63× water immersion objective lens at room temperature and the pinhole was set at 1 airy unit. Z-stack images of 0.75 μm thickness were acquired with the Zeiss Zen Image browser (Zeiss GmbH) using the “Multi Track” option of the microscope to exclude the cross-talk between the channels. Five to ten fields were imaged for each HD and HNSCC patient.

#### Image Analysis

Images were analyzed using ImageJ software (NIH, Bethesda, MD). Regions of interest (ROI) analysis: Briefly, in each field, the z-stack in the focal plane of the cell membrane (indicated by the presence of membrane localization of CD8 fluorescence) was visualized, and a ROI was drawn around the cell membrane and another ROI was drawn in the cytoplasmic region of individual T cells. The image threshold and background were adjusted and mean fluorescence intensities (mean gray values) for the CaM channels were measured for the membrane and cytoplasmic ROIs. The ratios of CaM fluorescence in the membrane and cytoplasmic ROIs were calculated for individual cells. For the experiments where cells were stained for KCa3.1, fluorescence intensity of KCa3.1 in HNSCC and HD T cells was measured in ROIs drawn around the cell membranes of individual cells.

Membrane and intracellular fluorescence measurement analyses of KCa3.1 and CaM were performed by comparing mean gray values (fluorescence intensity)-distance (μm) profiles (line scans) on acquired images of HNSCC and HD cells. The z-stacks in the focal plane of the cell membrane were identified on basis of distinct membrane KCa3.1 fluorescence. Two to three lines were drawn across each cell, and linescan analysis was performed to identify KCa3.1 and CaM expression using the plot profile function on ImageJ. The abscissa corresponding to outermost KCa3.1 fluorescence peaks on the linescan plot defined the cell surface, where the channel was present. Fluorescence intensities (gray values) for KCa3.1 and CaM at the cell membrane (the points of KCa3.1 fluorescence peaks) were measured in every linescan analysis performed.

### Proximity Ligation Assay (PLA)

PLA experiments were performed using Duolink *In Situ* Detection Reagents Orange (DUO92102, Millipore Sigma) as described by us previously (Hajdu et al., 2015). Activated CD8^+^ T cells from HDs and HNSCC patients were seeded on poly-L-lysine-coated coverslips, fixed with 4% paraformaldehyde, permeabilized with PBS + 0.2% Triton X-100, blocked with PLA blocking solution (Millipore Sigma) for 1 h, and stained with mouse anti-human KCa3.1 (Clone 6C1, Alomone labs) and rabbit anti-human CaM antibodies (clone SJ16-09, Novus Biologicals) diluted in antibody dilution buffer (Millipore Sigma) for 3 h at room temperature. The specificity of the monoclonal rabbit anti-human CaM antibody is shown in **Figure S2**. PLA procedure was done according to the manufacturer's protocol. After the final washes, the cells were labeled with Alexa Fluor 647-conjugated rabbit anti-human CD8 alpha antibody (clone EP1150, Abcam) diluted in antibody dilution buffer for 2 h at room temperature. The coverslips were washed, stained with 4′, 6-diamidino-2-phenylindole (DAPI, 1 μg/ml, ThermoFisher) to label the cell nuclei and mounted on glass slides as described earlier. Confocal images were obtained on a Zeiss LSM 710 microscope with 100× oil immersion lens at room temperature and the pinhole was set at 1 airy unit. Z-stack images of 0.75 μm thickness were acquired with the Zeiss Zen Image browser (Zeiss GmbH) using the “Multi Track” option of the microscope to exclude the cross-talk between the channels. Five to ten fields were imaged for each HD and HNSCC patient. Successful interaction by PLA between the KCa3.1 and CaM pairs were detected as bright fluorescent spots. As technical controls for non-specific binding, PLA was performed by omitting either the KCa3.1 or CaM antibodies or PLA probes (**Figure S3**).

Quantitation of PLA signals in the acquired images was performed using ImageJ. Briefly, the image threshold and background were adjusted for each image and a ROI was drawn around the cell membranes (identified by CD8 staining) and the positive PLA signals (dots) within the ROI were quantified by particle analysis, normalized to the number of cells counted in each field, and presented as the number of dots counted per cell membrane. For three-dimensional visualization of the PLA signal on the membrane, the z-stacks from representative PLA confocal images of T cells from HD and HNSCC individuals acquired as described earlier were reconstructed into 3D using Imaris image analysis software (Bitplane AG, Zurich, Switzerland).

### Electrophysiology

KCa3.1 and Kv1.3 currents were recorded from activated CD8^+^ T cells in whole-cell voltage clamp configuration using an AxoPatch 200B Amplifier (Molecular devices). Pipettes were formed from Borosilicate glass (TW150F-4, World Precision Instruments) with a P-97 horizontal puller (Sutter Instruments) and had a resistance between 4 and 5 MΩ when filled with intracellular pipette solution consisted of (mM) 145 K-Aspartate, 2 MgCl_2_, 8.5 CaCl_2_, 10 EGTA, and 10 HEPES. The pH value was set to 7.2 using TRIS buffer. Unless stated otherwise 1 µM free Ca^2+^ concentration was maintained inside the pipette solution. The external solution consisted of (mM) 160 NaCl, 4.5 KCl, 1 MgCl_2_, 10 HEPES; the pH value was set to 7.4 using NaOH. In a subset of experiments we utilized pipette solution with 3 and 10 μM free Ca^2+^ concentration. Free Calcium concentration was calculated using a program developed by Stanford University. (http://web.stanford.edu/~cpatton/webmaxcS.htm).

Data were acquired using pCLAMP 8.0 software (Molecular Devices) through a 16-bit A-D/D-A interface (Digidata1320A, Molecular Devices). Data were low pass filtered at 2 kHz and digitalized at 100 kHz. The electrophysiological protocol to record KCa3.1 currents implemented a ramp pulse from −120 to +50 mV from a holding potential of −70 mV every 15 s. The macroscopic conductance (G) of KCa3.1 channels was calculated as a ratio of the liner fraction of macroscopic current slope to the slope of ramp voltage stimulus after subtraction of the leak current (Chimote et al., 2018). The KCa3.1 slope conductance was calculated using linear regression in the voltage range between −100 and −80 mV to avoid contamination by the Kv1.3 current. Kv1.3 currents were determined from the same ramp protocol at +50 mV after subtraction of the KCa3.1 current extrapolated by linear regression. CaM was dissolved in pipette solution to make a final concentration of 50 µM. While performing electrophysiology experiments on HD T cells transfected with either si*CALM1* or scr-RNA, the cells expressing GFP fluorescence were evaluated.

### Intracellular Ca^2+^ Measurements

CD8^+^ T cells from HD or HNSCC patients were plated on poly-L-lysine coated coverslips, loaded with 1 μM of Fura-2/AM (ThermoFisher) and Fura-2 intensities were measured using InCytIm2 Ca^2+^ imaging system (Intracellular Imaging, Cincinnati, OH, USA) as previously described (Robbins et al., 2005; Kuras et al., 2012). Briefly, the cells were first perfused with a 0 mM Ca^2+^ solution for 5 min, then they were perfused for additional 5 min with the 0 mM Ca^2+^ solution containing 1 μM thapsigargin (MilliporeSigma) and lastly, cells were perfused with 0.5 mM Ca^2+^ solution for 10–15 min. The 0 mM Ca^2+^ solution contained (in mM): 155 NaCl, 4.5 KCl, 1 MgCl_2_, 10 HEPES, 10 glucose, 2 EGTA, pH 7.4; while the 0.5 mM Ca^2+^ solution contained (in mM): 155 NaCl, 4.5 KCl, 2.5 MgCl_2_, 10 HEPES, 10 glucose, 0.5 CaCl_2_, pH 7.4. A standard curve was used to correlate the measured ratiometric Fura-2 intensities (340/380 nm ratio) to the intracellular Ca^2+^ levels, as per the manufacturer's instructions. Change in Ca^2+^ (ΔCa^2+^ flux) was calculated as the difference in the measured Fura-2 ratio from its peak value in the presence of 0.5 mM Ca^2+^ to the baseline measured after addition of thapsigargin.

### siRNA

Silencer^®^ Pre-designed siRNA against *CALM1* gene (siRNA ID 146695, catalog number AM51331) and scrambled sequence siRNA (Silencer^®^ Negative Control #1 siRNA, catalog number AM4611) were obtained from ThermoFisher.

### Transfection

1–6 × 10^6^ CD8^+^ T cells that were isolated from HD and activated for 72 h were transfected using the Amaxa™ P3 Primary Cell 4D-Nucleofector™ X Kit (Lonza Cologne GmbH). 100 nM si*CALM1* or scr- RNA were cotransfected with 2 μg GFP (pMAX GFP supplied in the Amaxa Nucleofector Kit) using program E0-115 (program: “T cell stimulated”) using a 4D-Nucleofector™ unit (Lonza Cologne GmbH) as per manufacturer's instructions. The transfected cells were incubated overnight at 37° C and further activated for 24 h with anti-CD3 and anti-CD28 antibodies (total 96 h activation). The efficiency of transfection was ~30% as determined by cells expressing GFP fluorescence. The cells that expressed GFP were considered to be transfected successfully and were used for electrophysiology, flow cytometry, and chemotaxis experiments.

### Flow Cytometry

CD8^+^ T cells (~1 × 10^6^ cells per condition) were stained with Zombie Aqua Fixable Viability Kit (Biolegend) according to manufacturer's instructions, fixed with 4% paraformaldehyde (Affymetrix, Thermo Fisher Scientific), stained with ATTO 488–conjugated mouse anti-human KCa3.1 extracellular antibody (clone 6C1, Alomone Labs) without permeabilization. For staining with CaM, fixed cells were permeabilized with 0.1% Triton X-100 (MilliporeSigma) and stained with Alexa Fluor 647–conjugated mouse anti-human CaM antibody (clone 2D1, Novus Biologicals). To test for T cell activation, CD8^+^ T cells were stained live with APC-Fire™750–conjugated mouse anti-human anti-CD69 antibody (Clone FN50, Biolegend) and then fixed with 1% paraformaldehyde. For intracellular IFNγ staining, PBMC were isolated from HD and were stimulated for 24 h with cell activation cocktail (Biolegend) and were transfected with either si*CALM1* or scr-RNA along with GFP as described earlier. Post-transfection, cells were stimulated for 3 h with cell activation cocktail in the presence of brefeldin A (BD Biosciences) in the presence or absence of 1-EBIO. We also isolated PBMC from HD's and HNSCC individuals and stimulated them for 3 h in the presence of brefeldin A in the presence or absence of 1-EBIO. Unstimulated HD and HNSCC PBMC were also incubated in the presence of brefeldin A. All the cells were first stained with Zombie Aqua Fixable Viability Kit according to the manufacturer's instructions, surface stained with Pacific Blue conjugated mouse-anti human anti-CD3 antibody (Clone OKT3, Biolegend), fixed and permeabilized using BD Cytofix/Cytoperm kit (BD Biosciences). The permeabilized cells were then stained with Brilliant violet 605 conjugated mouse anti-human anti-IFNγ antibody (Clone 4S.B3, BioLegend). For all flow cytometry experiments, data were collected on an LSR II flow cytometer (BD Biosciences) and analyzed with FlowJo software (FlowJo LLC). In experiments performed on T cells transfected with si*CALM1* or scr-RNA, cells that were gated on the GFP positive population (indicative of successful transfection) were used for data evaluation.

### Chemotaxis

Three-dimensional chemotaxis was performed using the μ-Slide Chemotaxis assay (ibidi GmbH) as described previously (Chimote et al., 2018). Briefly, ~1 × 10^6^ CD8^+^ T cells were activated for 72 h and then transfected with either si*CALM1* or scr-RNA along with GFP and further activated for 24 h. The cells were then incorporated in a type I rat tail collagen gel (Corning) as per the manufacturer's instructions and added to the central observation chamber of the μ-Slide Chemotaxis. CXCL12 was used as the chemokine for all chemotaxis experiments and 8 μg/ml CXCL12 was added to the migration medium in the reservoir to the left of the observation chamber to generate a chemokine gradient to measure the baseline chemotaxis as described previously (Chimote et al., 2018). To assess the effects of adenosine on chemotaxis, we established simultaneous adenosine and chemokine gradients in a separate chamber as reported previously (Chimote et al., 2018). The slide was then mounted on the stage of an inverted Zeiss LSM 710 microscope (Carl Zeiss Microscopy GmbH) equipped with a 37°C incubator. Time-lapse video microscopy was performed to record cell migration and images were acquired every 3 s for up to 3 h in the bright field and GFP channels. Cells that were successfully transfected were identified by the presence of a strong intracellular GFP signal and cell tracking was performed using the “Manual Tracking plugin” on ImageJ software (National Institutes of Health), and the data were analyzed using the Chemotaxis and Migration Tool (ibidi GmbH). Ten to 15 transfected (GFP positive) cells were tracked per condition. Chemotactic effect was assessed by measuring the center of mass (COM, the average position along the relevant axis that the cells reached by the end of the experiment). A positive chemotaxis was defined if the cells migrated along the CXCL12 gradient (y axis) that is, a Y-COM > 0 indicated a positive chemotaxis (Chimote et al., 2018).

### Statistical Analysis

Statistical analyses were performed using Student's t test (paired or unpaired), Mann-Whitney rank sum test (in experiments where samples failed normality), and ANOVA as indicated. *Post hoc* testing on ANOVA was done by multiple pairwise comparison procedures using the Holm-Sidak method. Statistical analysis was performed using SigmaPlot 13.0 (Systat Software Inc.). P value of less than or equal to 0.05 was defined as statistically significant.

## Results

### Expression of Calmodulin Is Reduced in HNSCC CD8^+^ T Cells

**Figure 1:**
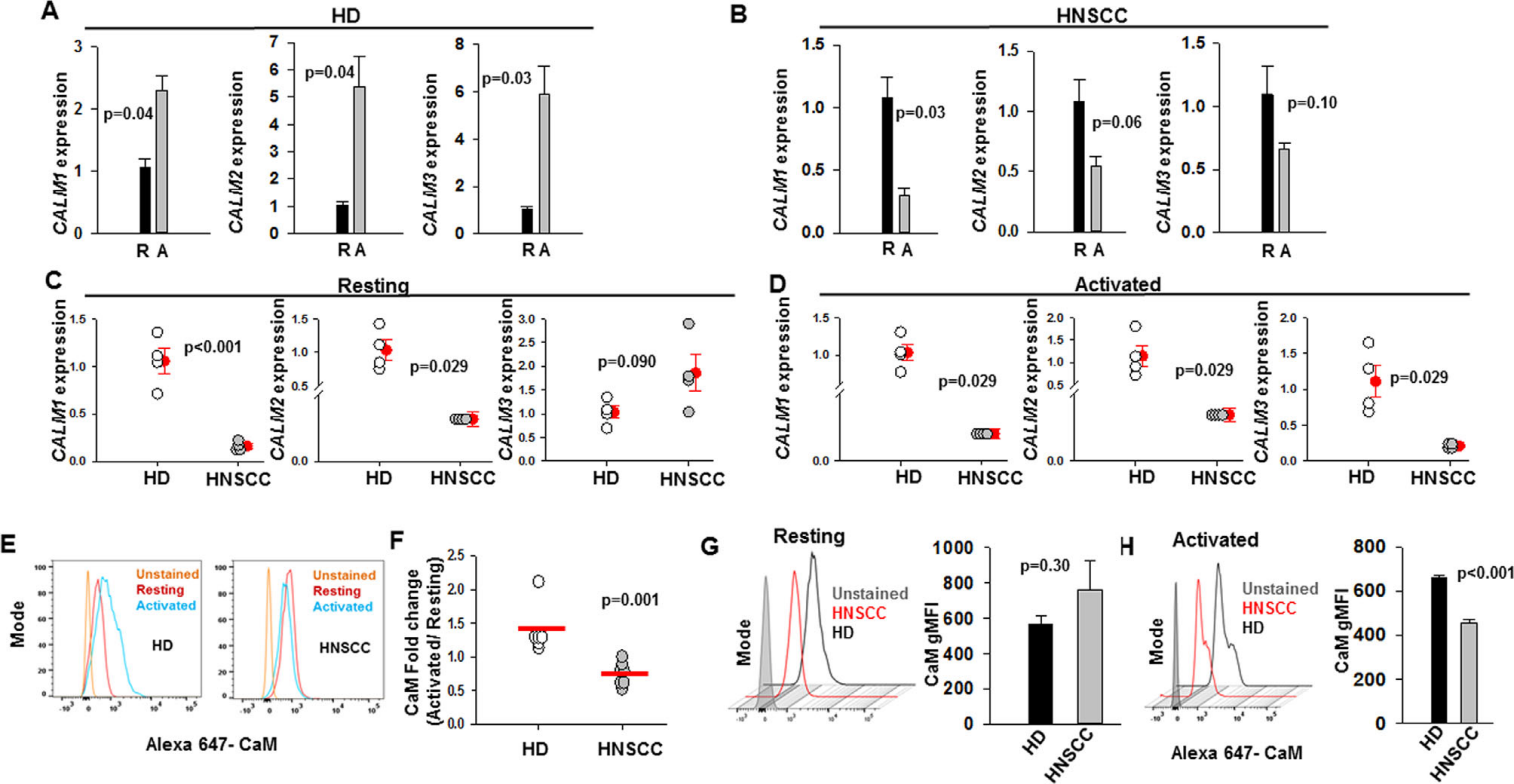
Decreased calmodulin expression in HNSCC T cells. **(A, B)** *CALM1*, *CALM2*, and *CALM3* expression in resting, “R” and activated, “A” T cells in HD (panel **A**) and HNSCC (panel **B**) patients were quantified by reverse transcription quantitative polymerase chain reaction (RT-qPCR). Data are the fold change in *CALM1*, *CALM2*, and *CALM3* expression relative to *18S rRNA* expression. The data were normalized to mean resting *CALM1*, *CALM2*, and *CALM3* gene expression in HD and HNSCC. For panels **(A, B)** bars represent mean ± SEM from four HD and four HNSCC patients, with samples run in quadruplicate. **(C, D)** *CALM1*, *CALM2*, and *CALM3* expression in resting **(C)** and activated **(D)** T cells in HD and HNSCC patients were quantified by RT-qPCR. Data are the fold change in *CALM1*, *CALM2*, and *CALM3* expression relative to *18S rRNA* expression. The data were normalized to mean *CALM1*, *CALM2*, and *CALM3* expression in HD T cells for the resting and activated conditions. For panels **(C, D)** the solid red circles and error bars represent mean ± SEM of four HD and four HNSCC patients, with samples run in quadruplicate. **(E)** Flow cytometry histograms showing CaM expression in resting and activated T cells from representative HD and HNSCC patients. **(F)** Fold change in CaM geometric mean fluorescence intensity (gMFI) in T cells from HD (n = 6) and HNSCC patients (n = 7) calculated as a ratio of CaM gMFI in activated cells over CaM gMFI in resting cells. Horizontal red line represents the mean for each group. **(G)** Representative flow cytometry overlay histogram showing CaM expression in resting T cells from one HD and one HNSCC patient. The bar graph on the right shows gMFI for CaM expression in resting T cells from HD (n = 6) and HNSCC patients (n = 7). Bars represent mean ± SEM. **(H)** Representative flow cytometry overlay histogram showing CaM expression in activated T cells from one HD and one HNSCC patient. CaM expression was measured in live cell population identified by exclusion from Zombie Aqua live/dead stain. The bar graph on the right shows gMFI for CaM expression in activated T cells from HD (n = 3) and HNSCC patients (n = 3). Data in **(A, B)** were analyzed by paired student's t-test; data in **(C)** for *CALM1* and *CALM3* expression were analyzed by student's t-test, whereas data for *CALM2* expression were analyzed by Mann-Whitney rank sum test, data in **(D, F)** were analyzed by Mann Whitney rank sum test; data in **(G, H)** were analyzed by Student's t-test.

Experiments were performed to assess whether CaM expression is altered in CD8^+^ T cells of HNSCC patients. CaM is a small protein of 148 amino acids (~17kD), which is encoded by three genes *CALM1*, *CALM2*, and *CALM3*, and downregulation of either of these genes can result in decreased CaM protein levels (Colomer et al., 1993; Marshall et al., 2015). We investigated CaM mRNA and protein levels in resting and activated T cells in healthy donors (HD) and HNSCC patients (**Figure 1**). All experiments were conducted on CD8^+^ T cells that were isolated from HD and HNSCC patients and activated *in vitro* using anti-CD3 and anti-CD28 antibodies for 72 to 96 h, unless otherwise specified. We have previously reported that there is no difference in the activation status of HNSCC and HD T cells (Chimote et al., 2018). KCa3.1 and CaM expression is increased in activated healthy T cells (Colomer et al., 1993; Ghanshani et al., 2000; Cahalan and Chandy, 2009). T cells from HD showed an increase in *CALM1*, *CALM2*, and *CALM3* expression on activation (**Figure 1A**), whereas HNSCC T cells failed to upregulate *CALM1*, *CALM2*, and *CALM3* mRNAs (**Figure 1B**). Essentially, *CALM1* and *CALM2* mRNA levels were significantly reduced in resting HNSCC T cells as compared to resting HD T cells, whereas *CALM1*, *CALM2*, and *CALM3* mRNA levels were significantly lower in activated HNSCC T cells as compared to their activated healthy counterparts (**Figures 1C, D**). In the qPCR experiments, we used *18s rRNA* as the housekeeping gene. Comparable results were obtained with *GAPDH* as the housekeeping gene (**Table S2**). Measurement of CaM protein levels by flow cytometry confirmed that HD T cells increased CaM post-activation, while CaM decreased after activation in HNSCC T cells (**Figures 1E, F**). CaM protein abundance was not significantly different in resting HD and HNCC T cells (**Figure 1G**), while in activated HNSCC T cells it was 31% lower than their healthy counterparts (**Figure 1H**).

### HNSCC T Cells Display a Compartmentalized Decrease in Membrane CaM

**Figure 2:**
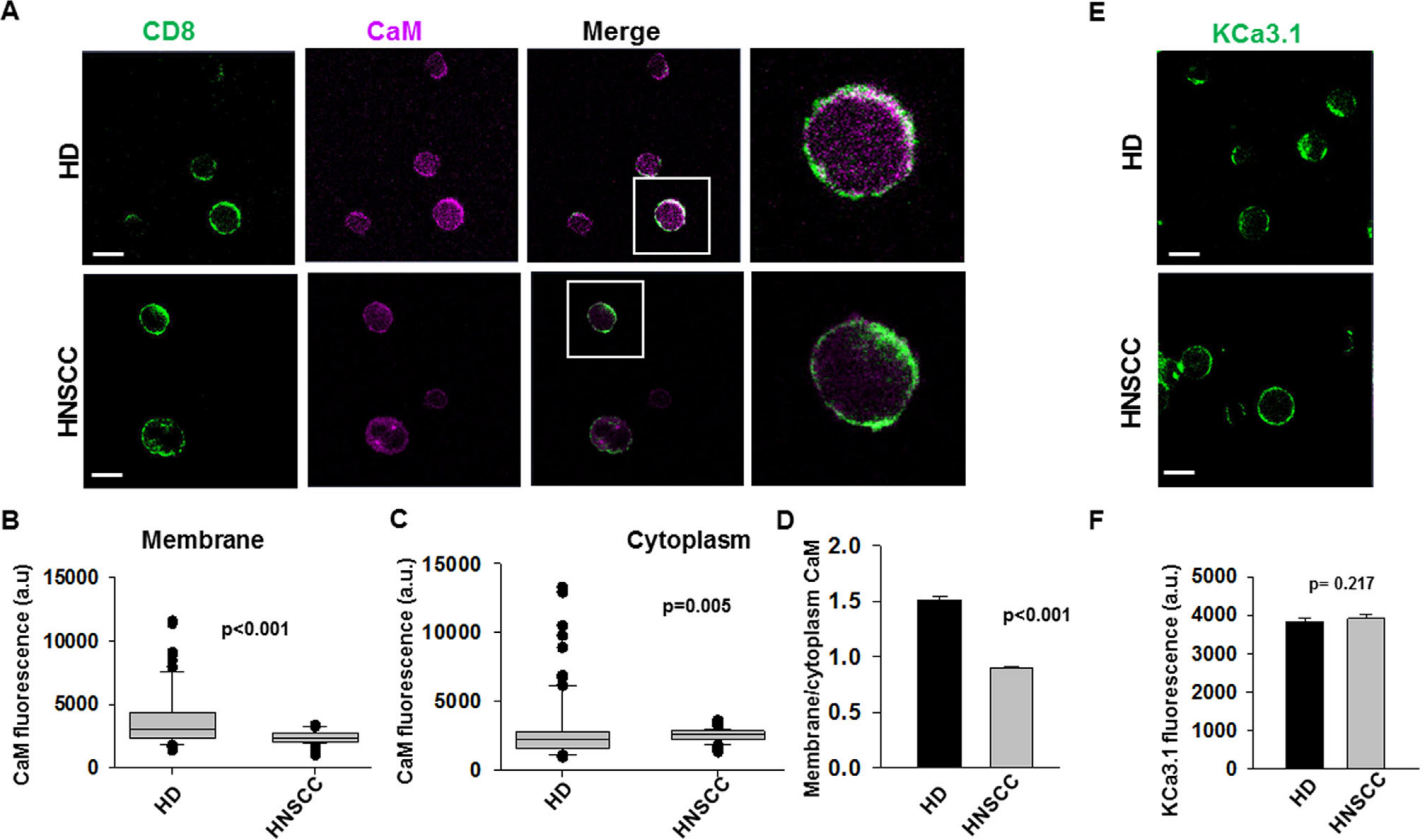
Decreased membrane CaM in HNSCC T cells. **(A)** Representative confocal images of HD and HNSCC T cells stained for CD8 (green; no permeabilization) and CaM (magenta, after permeabilization). Scale bars indicate 10 μm. Representative individual cells highlighted (box) in the merged images are magnified in the extreme right panels. Co-localization is indicated by white color. **(B, C)** Fluorescence intensities of CaM in the membrane **(B)** and cytoplasm **(C)** of HD and HNSCC T cells were measured as described in *Materials and Methods*. Data represented by a box and whisker plot. The horizontal line represents median value of CaM fluorescence intensities for each group, while the first and third quartiles are represented in the top and bottom boxes respectively. **(D)** Ratio of CaM fluorescence at the cell membrane and cytoplasm of HD and HNSCC T cells. Bars represent mean ± SEM **(E)** Representative confocal images of HD and HNSCC T cells stained for KCa3.1 (green). Scale bars indicate 10 μm. **(F)** Fluorescence intensities of KCa3.1 in the membranes of HD and HNSCC T cells. Bars represent mean ± SEM of 117 cells from 3 HD and 61 cells from 3 HNSCC patients. Shown in **(B–D)** are values for 96 cells from 3 HD and 110 cells from 3 HNSCC patients. Data in **(B–D, F)** were analyzed by Mann Whitney rank sum test.

A reduction in CaM could have enormous implications in T cell function as CaM oversees the activity of many molecules (Stocker, 2004; Berchtold and Villalobo, 2014; Lee and Mackinnon, 2018). Thus, we conducted experiments to determine whether the reduction of CaM in HNSCC T cells spanned all cell compartments. Confocal microscopy experiments were conducted in CD8^+^ T cells from HNSCC patients and from HD to determine the CaM membrane and cytoplasmic abundance. Cells were fixed, stained with anti-CD8 antibody to label the membrane (prior to permeabilization), and then, stained post-permeabilization with anti-CaM antibody (**Figure 2A**). We evaluated CaM levels in HD and HNSCC T cells by measuring the fluorescence intensity of CaM at the cell membrane (defined by the CD8 staining) as well as in the cytoplasm. The distribution of membrane CaM differs in HNSCC vs HD T cells (**Figures 2B, D**). Membrane CaM was lower in HNSCC T cells as compared to their healthy counterparts by ca. 41% (**Figure 2B**), while the cytoplasmic CaM was 13% higher in HNSCC T cells as compared to their healthy counterparts (**Figure 2C**). CaM levels in the membranes of HD T cells were 51% higher than in the cytoplasm (with a membrane: cytoplasm CaM fluorescence ratio of 1.5); in HNSCC T cells they were 10% lower (membrane: cytoplasm CaM fluorescence ratio of 0.9) (**Figure 2D**). On the other hand, activated CD8^+^ T cells from HD and HNSCC individuals stained with extracellular anti-KCa3.1 (with no permeabilization, **Figure 2E**) showed similar membrane abundance of KCa3.1, as reported previously (Chimote et al., 2018) (**Figure 2F**). Overall, these data indicate that HNSCC T cells express less CaM than HD T cells, and this difference is confined to the membrane compartment. This, in turn, suggests that in HNSCC T cells, fewer KCa3.1 channels may be coupled to CaM than in HD T cells. It also suggests that the defect in CaM of HNSCC T cells may not impact other functions that rely on cytoplasmic CaM.

### HNSCC T Cells Show Lower Association of Membrane KCa3.1 with CaM and Decreased KCa3.1 Activity

**Figure 3:**
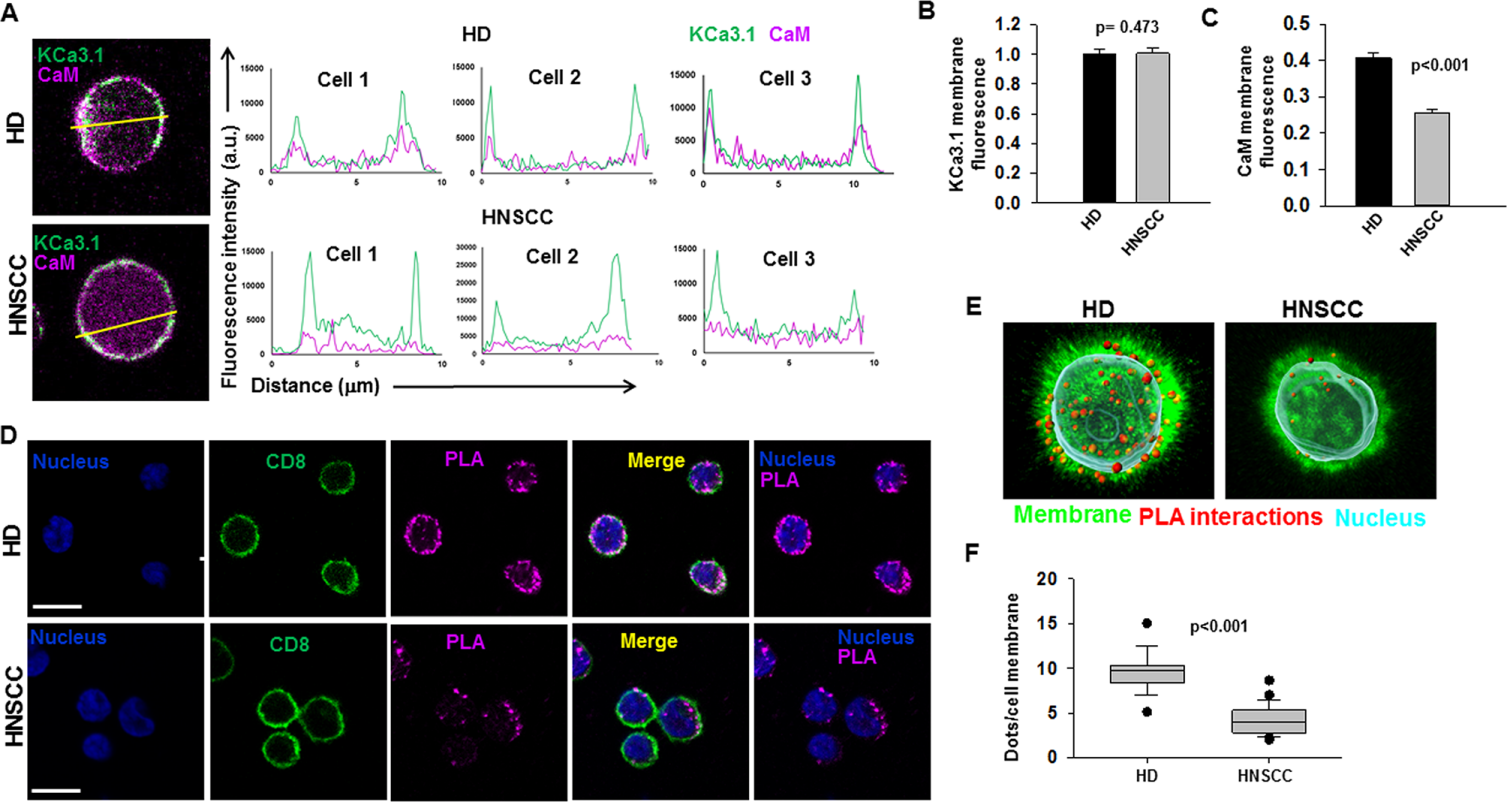
Reduced association of KCa3.1 with CaM in HNSCC T cells **(A)** Linescan analysis of KCa3.1 and CaM association at the cell membrane. (Left) Magnified representative confocal images of HD and HNSCC T cells stained for KCa3.1 (green) and CaM (magenta) from the merged images in **Figure S4** (box). Lines were drawn across the cells for linescan analysis, and representative lines drawn for a linescan are shown. (Right) Line scan analyses were generated using ImageJ software and are plotted as KCa3.1 (green line) and CaM (magenta line) fluorescence intensity (y-axis) *vs.* the length of the lines drawn across the merged cells (distance from the starting point of the line, μm, x-axis). Shown here are linescan graphs from three representative HD and HNSCC T cells, wherein the outmost KCa3.1 (green) peaks correspond to the cell membrane. Correspondence of the peaks for green (KCa3.1) and magenta (CaM) lines is indicative of their colocalization. **(B)** Comparison of KCa3.1 membrane expression in HD and HNSCC T cells. KCa.31 fluorescence intensities were measured at the cell surface (points corresponding to KCa3.1 fluorescence peaks on the cell membrane) in linescan plots **(A)**. The data were normalized to mean KCa3.1 fluorescence intensity measured in HD T cells. **(C)** Comparison of CaM membrane-associated expression in HD and HNSCC T cells. CaM fluorescence intensities were measured at the cell surface (points corresponding to KCa3.1 fluorescence peaks on the cell membrane) in linescan plots **(A)**. The data were normalized to mean KCa3.1 fluorescence intensity measured in HD T cells, which represents the proportion of CaM associated with membrane KCa3.1. For **(B, C)**, bars represent mean ± SEM for data from 108 points (corresponding to KCa3.1 fluorescence peaks in the linescans) analyzed in 18 cells from 3 HD, and 120 points analyzed in 20 cells from 3 HNSCC patients. **(D)** Interaction between KCa3.1 and CaM in cell membranes of HD and HNSCC T cells. Confocal images of representative cells from PLA experiments performed in HD (top) and HNSCC (bottom). Cells were incubated with rabbit anti-human CaM and mouse anti-human KCa3.1 antibodies, after which PLA probe–ligated secondary antibodies were then added and PLA was performed as described in *Materials and Methods*. Positive PLA signals showing single protein interactions between KCa3.1 and CaM are visualized as magenta fluorescent dots. Cell membranes are labeled with CD8 antibodies (green) and nuclei are labeled with DAPI (blue). Scale bar, 10 μm. **(E)** 3D views of the PLA dots (red) in relation to the nucleus (DAPI, cyan) and membrane (CD8, green) in a representative HD (left) and HNSCC (right) T cell. **(F)** Average PLA signals. PLA signals in the cell membranes of in HD and HNSCC T cells were quantified as number of dots per cell membrane and are represented as a box plot. The dots per cell membrane are reported as median (horizontal line), first (top box), and third quartiles (bottom box). Shown are values for 185 cells from 3 HD and 214 cells from 4 HNSCC patients. For **(B, C)**, significance was determined by Mann Whitney rank sum test, while the significance for **(F)** was determined by Student's t-test.

**Figure 4:**
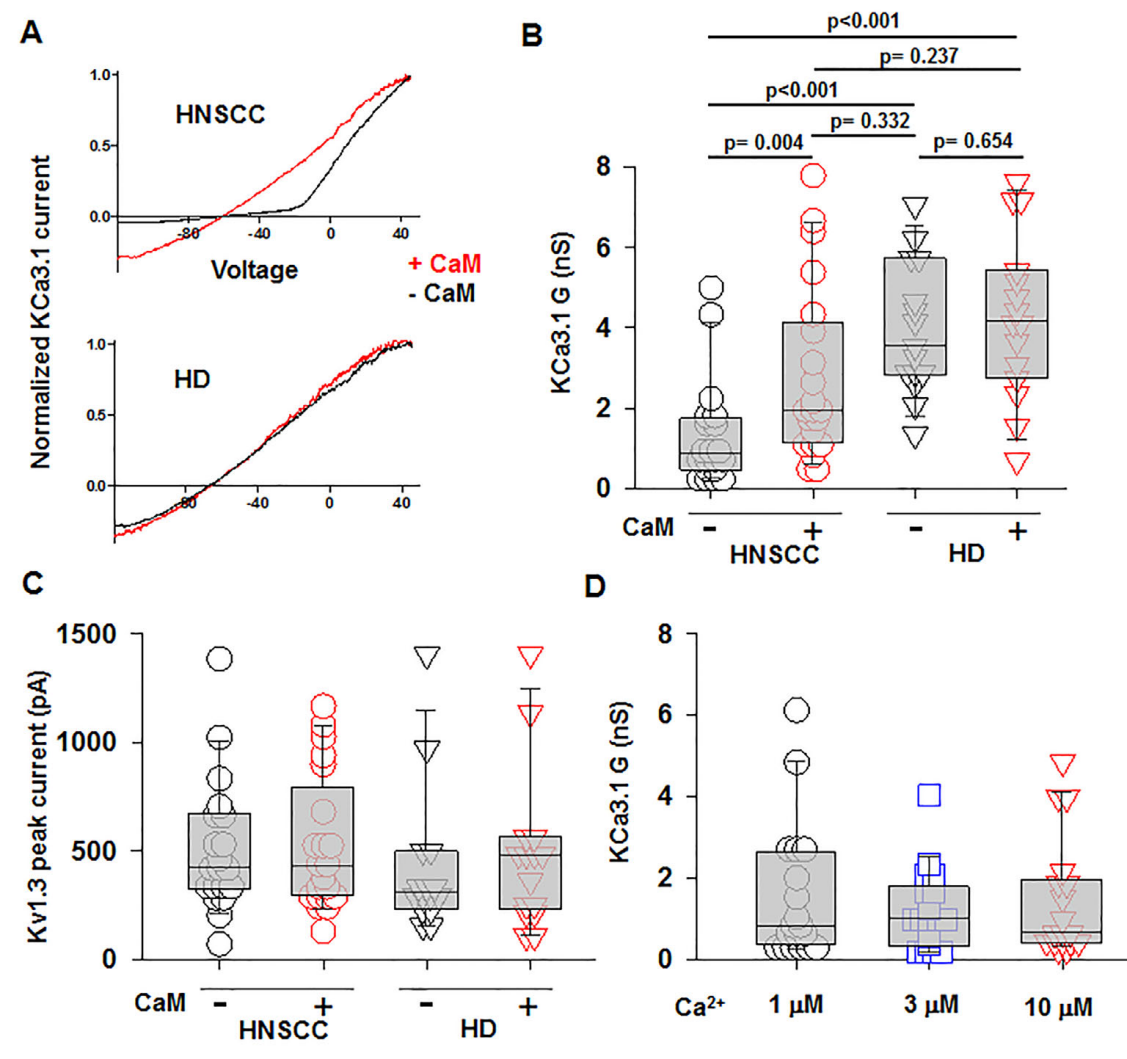
Increased KCa3.1 activity in HNSCC T cells by intracellular delivery of CaM. **(A)** Representative KCa3.1 currents recorded in whole-cell voltage-clamp configuration in activated T cells from a HNSCC patient and a HD in the presence or absence of 50 μM CAM. The KCa3.1 currents are normalized for the maximum current at +50 mV to ease comparison of the KCa3.1 conductance at hyperpolarizing voltages. **(B)** KCa3.1 conductance, G (Panel **C**) and Kv1.3 peak currents measured in the presence or absence of 50 μM CaM in T cells from HNSCC patients (n = 20 cells without CaM and n = 21 cells with CaM from 4 patients) and HD (n = 20 cells from three individuals). **(D)** KCa3.1 G measured in the presence 1 μM, 3 μM, and 10 μM free Ca^2+^ in the patch pipette in activated HNSCC T cells (n = 18 cells for each conditions from three patients). The values in panels B, C, and D are presented as box and whisker plots. The data are reported as the median (horizontal line), first (top box), and third quartiles (bottom box). The KCa3.1 G or Kv1.3 peak currents were measured in at least five cells for each condition in an individual. Data in **(B, C)** were analyzed by two-way ANOVA [**(B)**: p < 0.001 for HD vs HNSCC and p = 0.046 for + CaM *vs.* –CaM; **(C)**: p = 0.359 for HD *vs.* HNSCC and p = 0.673 for + CaM *vs.* –CaM], while data in **(D)** were analyzed by one-way repeated measures ANOVA (p = 0.796).

**Figure 5:**
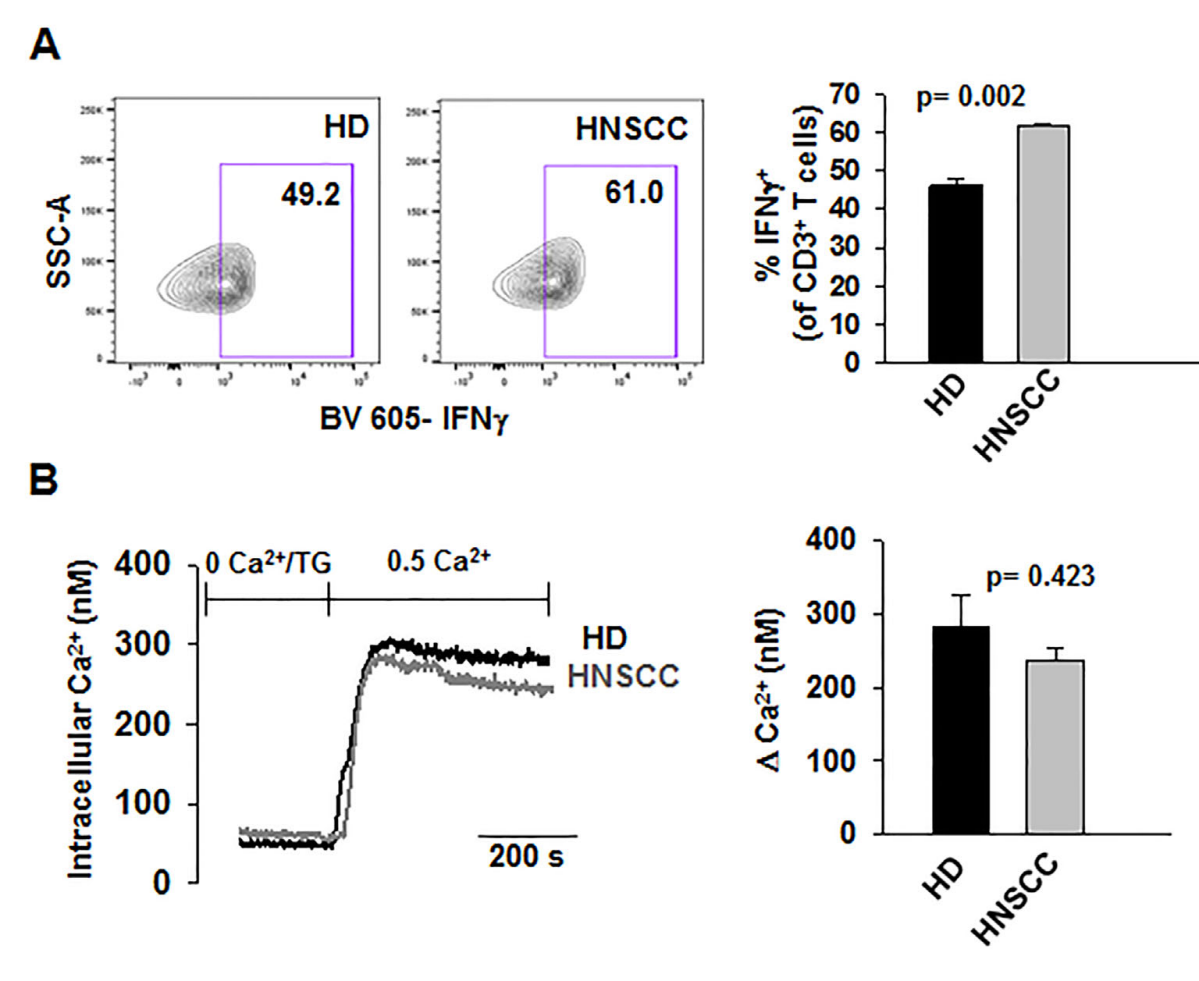
Ca^2+^- dependent T cell functions are not decreased in HNSCC T cells **(A)** Representative flow cytometry plots gated on stimulated CD3^+^ T cells from a HD and HNSCC patient showing IFNγ production. (Right) Quantification of IFNγ-production in stimulated HD and HNSCC T cells. Cells were stimulated as described in *Materials and Methods*. Live cell population was identified by exclusion from Zombie Aqua live/dead stain and gated on CD3^+^ T cell population. IFNγ^+^ T cell populations were selected by drawing rectangle gates. The numbers within the gates are the percentages of IFNγ^+^ CD3^+^ T cells. **(B)** (Left) Representative Ca^2+^ response (presented as intracellular Ca^2+^ levels) recorded in a HD and HNSCC patient. Cells were loaded with Fura-2 and intracellular fluorescence was recorded and quantified using a standard curve as described in *Materials and Methods*. (Right) Average change in intracellular Ca^2+^, expressed as ΔCa^2+^, the difference between the recorded peak Ca^2+^ and baseline Ca^2+^ (see *Materials and Methods*) in HDs and HNSCC patients. In **(A, B)**, bars represent mean ± SEM from experiments performed in three HDs and HNSCC patients. Data were analyzed by Student's t-test.

We performed confocal microscopy experiments to measure the association of CaM with KCa3.1 at the plasma membrane. CD8^+^ T cells from HD and HNSCC individuals were stained with extracellular anti-KCa3.1 (with no permeabilization) and intracellular anti-CaM (after permeabilization) antibodies (**Figure S4**). The membrane distribution of KCa3.1 and CaM was determined by linescan analysis. We observed that in the membranes of HNSCC cells, the fluorescence intensity of CaM (**Figure 3A**, magenta) corresponding to the KCa3.1 peaks (green), was lower than that in HD T cells. In some HNSCC T cells, we observed KCa3.1 proteins with no, or undetectable, associated CaM. Indeed, while KCa3.1 peak fluorescence intensities at the membrane were comparable in HD and HNSCC T cells, there was a 37% reduction in membrane KCa3.1-associated CaM (CaM fluorescence intensity corresponding to the KCa3.1 fluorescence intensity peaks, see *Materials and Methods*) in HNSCC T cells (**Figures 3B, C**). We then performed proximity ligation assay (PLA) to confirm whether the physical membrane association between CaM and KCa3.1 channels was reduced in HNSCC T cells. This assay detects the interaction between proteins that reside ≤ 40-nm through species-specific antibodies, each labeled with unique short oligonucleotides, which amplify when the two antibodies are in close proximity. The interaction is detected with a fluorescently labeled oligonucleotide that hybridizes to the amplicons, which can be subsequently visualized and quantified as discrete dots (PLA signals) by microscopy (Hajdu et al., 2015). As shown in **Figure 3D**, both HNSCC and HD T cells showed positive PLA signals (dots) at the cell membrane. However, analysis of the confocal images revealed that the number of dots/cell membrane in HNSCC T cells was reduced by 57% as compared to HD T cells (**Figures 3E, F**). These data confirm that KCa3.1 channels in the membranes of HNSCC T cells have a lower association with CaM. We performed further experiments to assess whether the decreased KCa3.1 function that we observed in HNSCC CD8^+^ T cells is due to decreased availability of CaM (Chimote et al., 2018). Patch-clamp experiments confirmed that KCa3.1 activity (defined by the whole-cell KCa3.1 conductance, G) was significantly lower in activated HNSCC T cells as compared to their healthy counterparts as previously shown (**Figures 4A, B**) (Chimote et al., 2018). However, intracellular addition of CaM, *via* the patch pipette, increased the KCa3.1 activity of HNSCC T cells to the level of HD T cells (**Figures 4A, B**). Conversely, we observed that addition of CaM did not increase KCa3.1 activity in HD T cells (**Figures 4A, B**). Addition of CaM had no effect on Kv1.3 activity (defined as peak current) in either HNSCC or HD T cells (**Figure 4C**). There was no change in cell capacitance (a measure of cell size) in the controls or CaM-treated cells either in HNSCC or HD T cells (**Table S3**). All patch-clamp experiments were performed with an internal pipette solution containing 1 μM free-Ca^2+^. To assess whether a decrease in Ca^2+^ sensitivity of the channels also contributed to the decrease in KCa3.1 function in HNSCC T cells, we measured KCa3.1 conductance in HNSCC T cells in the presence of incremental intracellular free Ca^2+^ concentrations (1 μM, 3 μM, and 10 μM). There was no effect of intracellular Ca^2+^ on the KCa3.1 conductance in HNSCC T cells (**Figure 4D**). Thus, these findings indicate that the decreased KCa3.1 activity in HNSCC T cells, which limits tumor infiltration, is due to an intrinsic defect in CaM (Chimote et al., 2018). We then investigated whether this defect in CaM observed in HNSCC T cells inhibits other Ca^2+^ and CaM-dependent functions like IFNγ production. We found no decrease in INFγ production in activated HNSCC T cells (**Figure 5A**). We also found no alterations in their Ca^2+^ fluxing abilities (**Figure 5B**) which, combined with the intact cytoplasmic CaM (**Figure 2C**), support an efficient IFNγ production. Overall, these data indicate that the alteration in CaM of HNSCC T cells selectively impacted T cell functions that depend on KCa3.1 activity.

### Knockdown of CALM1 in HD T Cells Reduces KCa3.1 Activity and Increases Their Sensitivity to Adenosine

**Figure 6:**
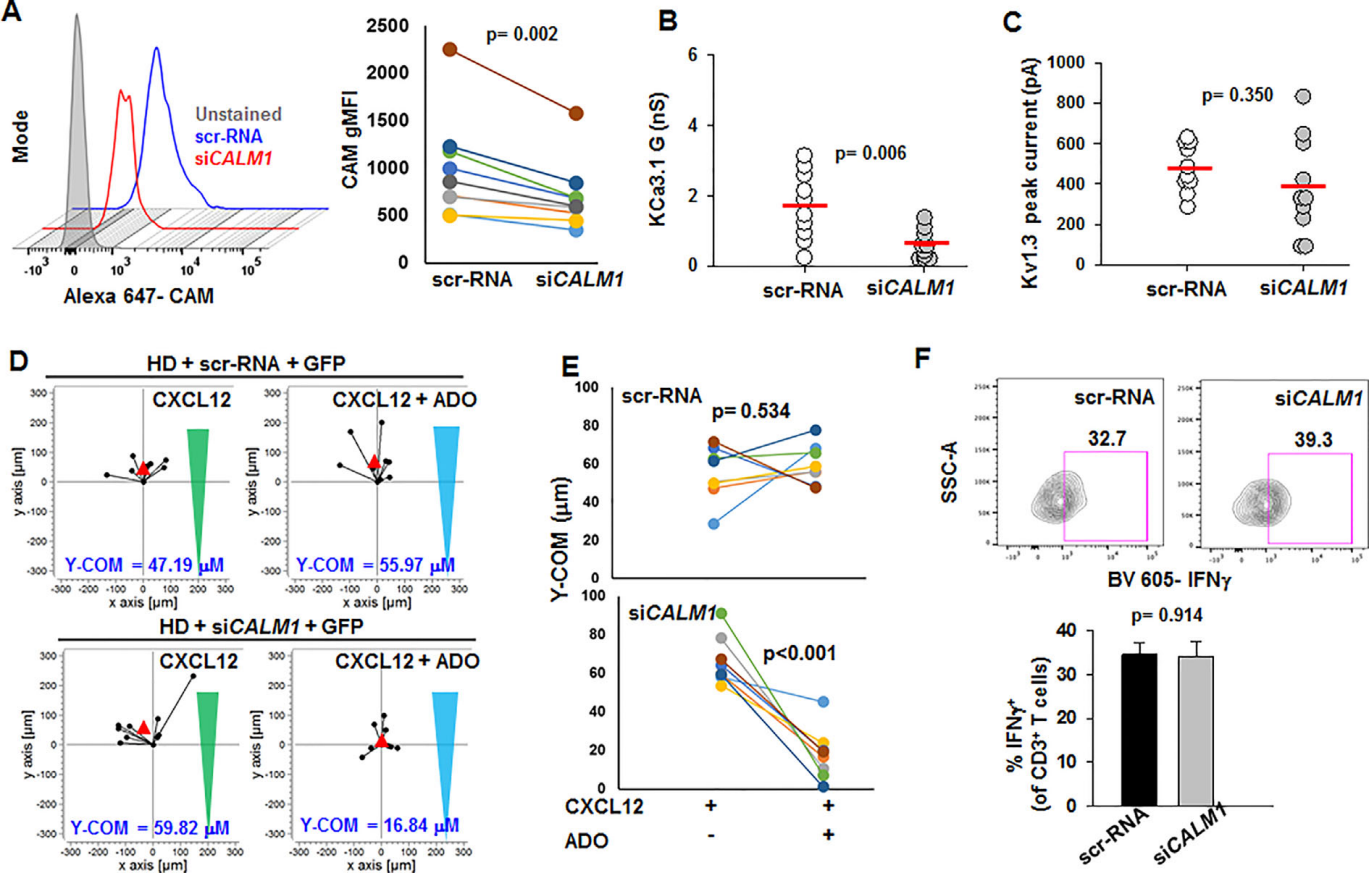
Reduction of KCa3.1 activity and increased adenosine sensitivity in HD T cells by *CALM1* knockdown. (**A**, left) Representative flow cytometry overlay histogram showing CaM expression in activated T cells from one HD transfected with scr-RNA and si*CALM1*. (Right) gMFI for CAM expression in T cells from nine HD transfected with scr-RNA and si*CALM1*. Transfected cells were identified by positive staining for GFP. **(B, C)** KCa3.1 conductance **(B)** and Kv1.3 peak currents **(C)** in T cells transfected with either si*CALM1* (n = 10 cells) or scr-RNA (n = 10 cells) from two HDs. Transfected cells were identified by positive staining for GFP. Horizontal red line represents mean values for each group. **(D)** Trajectories of T cells co-transfected with GFP and either scr-RNA or si*CALM1* migrating along either a CXCL12 gradient (green triangles) or a combination gradient of CXCL12 and adenosine (blue triangles) in a representative HD. Trajectories of GFP positive cells (indicating successful transfection) are shown for each condition. The starting point for each trajectory is artificially set to the same origin. The red triangles represent Y-COM. **(E)** Y-COM values for cells migrating along either a CXCL12 gradient or a combination gradient of CXCL12 with adenosine in HD T cells transfected with scr-RNA (n = 8 donors) and si*CALM1* (n = 8 donors). **(F)** (Left) Representative flow cytometry plots gated on CD3^+^ T cells transfected with either scr-RNA or si*CALM1* showing IFNγ production after stimulation. Live cell population was identified by exclusion from Zombie Aqua live/dead stain and gated on CD3^+^ population and transfected cells were identified by positive staining for GFP. IFNγ^+^ T cell populations were selected by drawing rectangle gates. The numbers next to the gates are the percentages of IFNγ^+^ CD3^+^ T GFP^+^ cells. (Right) Quantification of IFNγ induction in scr-RNA or siCALM1 transfected CD3^+^ T cells post 48 h activation in three HDs. Bars represent mean ± SEM. All data were analyzed by paired Student's t-test.

We then conducted experiments to study whether lowering CaM expression in HD T cells would result in a phenotype comparable to that of HNSCC T cells. We knocked down the *CALM1* gene in activated CD8^+^ T cells from HD using a siRNA against *CALM1* (si*CALM1*) while scrambled sequence siRNA (scr-RNA) was used as control. Transfection with si*CALM1* decreased CaM expression in activated HD T cells by 30% as compared to scr-RNA-transfected cells (**Figure 6A**); a reduction in CaM expression comparable to that observed in activated HNSCC T cells (**Figure 1H**). Patch-clamp experiments showed that si*CALM1* reduced KCa3.1 activity by 30% (**Figure 6B**), while Kv1.3 activity and cell capacitance remained unchanged (**Figure 6C**, **Table S3**). Silencing *CALM1* did not significantly decrease KCa3.1 and CD69 (T cell activation marker) protein levels (**Figure S5**). Thus, silencing *CALM1* reproduced in HD T cells the K^+^ channel phenotype previously reported for HNSCC T cells: low KCa3.1 activity, and normal Kv1.3 activity (Chimote et al., 2018). We conducted further experiments to see whether the knockdown of *CALM1* in HD T cells and the consequent decrease in their KCa3.1 function compromised other T cell functions. Low KCa3.1 activity has been implicated in the increased sensitivity of HNSCC T cells to adenosine that led to a heightened inhibitory effect of adenosine on chemotaxis (Chimote et al., 2018). Hence, we conducted 3D chemotaxis experiments to measure the effect of adenosine in si*CALM1*-transfected HD T cells (scr-RNA-transfected HD T cells were used as controls) using the methodology described previously (Chimote et al., 2018). We measured CXCL12-mediated 3D chemotaxis in si*CALM1* or scr-RNA-transfected HD T cells in the presence or absence of a concomitant chemokine and adenosine gradient. A representative experiment is shown in **Figure 6D**. Y-COM (*y-*coordinate of the center of mass, i.e., the *y*-coordinate of the averaged position the cells achieved at the end of the chemotaxis experiment; red triangles in **Figure 6D**) was used as a parameter to evaluate the chemotactic response. We observed that HD T cells transfected with scr-RNA migrated towards the chemokine even in the presence of adenosine (**Figure 6E**). However, when we knocked down *CALM1*, the transfected HD T cells lost their ability to chemotax in the presence of adenosine, as indicated by a decrease in their Y-COM (**Figure 6E**). We also found no alterations of IFNγ production in si*CALM1* transfected HD T cells (**Figure 6F**). Overall, these data indicate that the functional phenotype of HNSCC T cells is reproduced in HD T cells when CaM abundance is decreased (Chimote et al., 2018). Based on these findings, we tested whether, like in HNSCC T cells, KCa3.1 activators could be used to restore the channel's activity in si*CALM1*-transfected HD cells.

### Activation of KCa3.1 Potentiates the Chemotaxis of *CALM1*-Knockdown HD T Cells in the Presence of Adenosine

**Figure 7:**
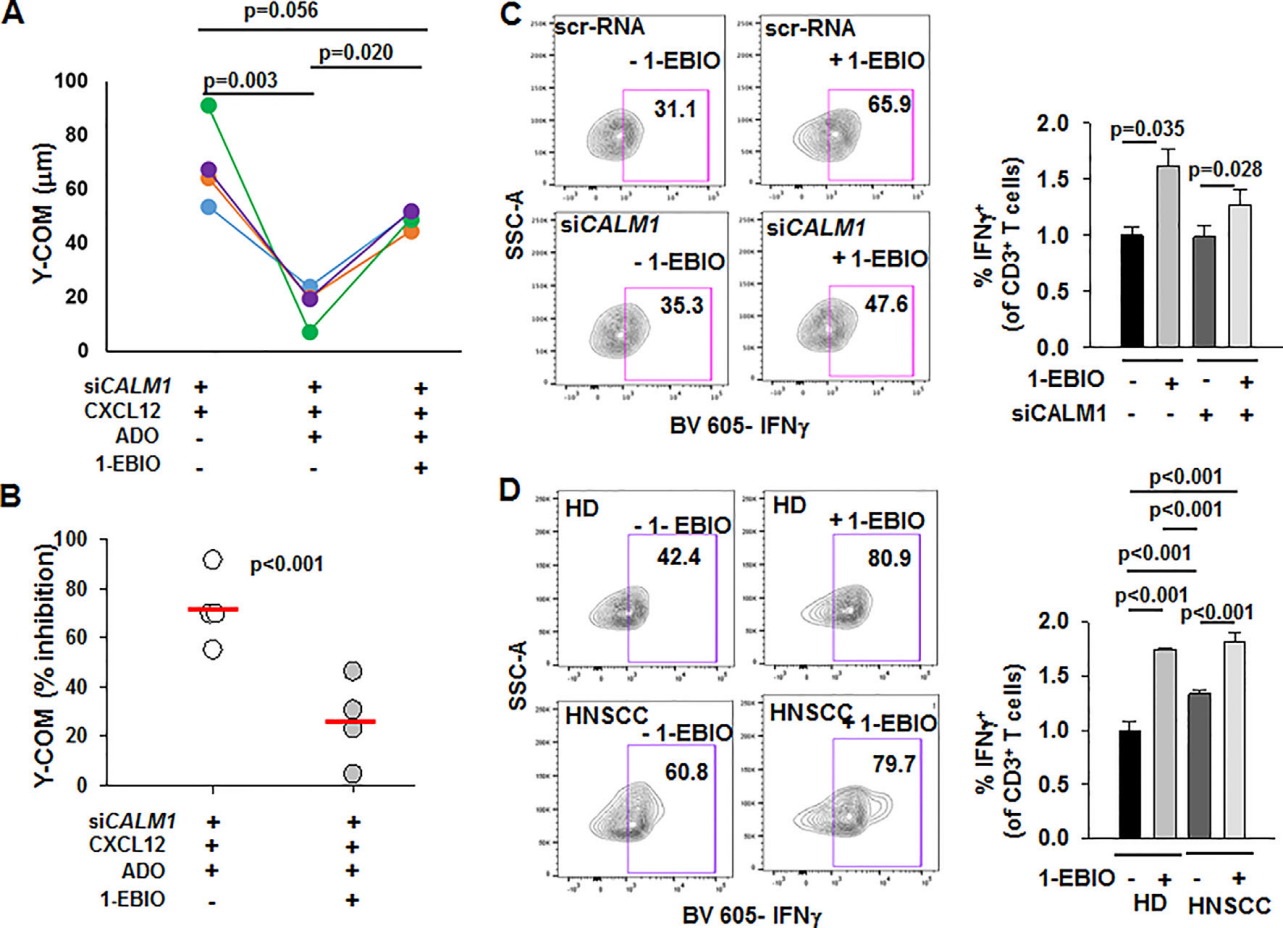
Potentiation of chemotaxis in the presence of adenosine and INFγ production by 1-EBIO in *CALM1*-knockdown HD T cells. **(A)** Y-COM values calculated for HD T cells transfected with si*CALM1* migrating along either a CXCL12 gradient or a combination gradient of CXCL12 with adenosine (ADO) with or without preincubation with 20 μM 1-EBIO (n = 4 donors). Transfected cells, identified by positive GFP staining, were tracked for analyzing Y-COM. **(B)** Percentage inhibition by ADO of Y-COM **(B)** in cells pretreated with 1-EBIO. Horizontal red line represents mean values for each group. **(C)** (Left) Representative flow cytometry plots gated on CD3^+^ T cells transfected with either scr-RNA or si*CALM1* with or without 1-EBIO (100 μM) showing IFNγ production after stimulation. Transfected cells were identified by positive staining for GFP. (Right) Quantification of IFNγ induction in scr-RNA or si*CALM1* transfected CD3^+^ T cells with or without 1-EBIO in three HD. The data were normalized to IFNγ production in control scr-RNA transfected HD cells. Bars represent mean ± SEM. **(D)** (Left) Representative flow cytometry plots gated on HD or HNSCC CD3^+^ T cells with or without 1-EBIO (100 μM) showing IFNγ production after stimulation. (Right) Quantification of IFNγ-producing HD and HNSCC T cells after stimulation in the presence or absence of 1-EBIO. The data were normalized to IFNγ production in control HD cells. For the HD + 1-EBIO condition, bars represent mean ± range for two individuals, while the rest of the bars in the graph represent mean ± SD for data from three HD and HNSCC patients. For analysis of **(C, D)**, live cell population was identified by exclusion from Zombie Aqua live/dead stain and gated on CD3^+^ population and transfected cells were identified by positive staining for GFP (in panel **C**). IFNγ^+^ T cell populations were selected by drawing rectangle gates. The numbers in the gates are the percentages of IFNγ^+^ CD3^+^ T cells. Data in **(A)** were analyzed with one-way repeated measures ANOVA (p <0.001), in **(B)** with paired Student's t test, and in **(C)** with a two-way ANOVA [p = 0.207 for scr-RNA *vs.* si*CALM1* and p = 0.006 for – 1-EBIO vs. +1-EBIO]. Data in **(D)** were analyzed by Kruksal-Wallis One Way ANOVA on Ranks (p < 0.001).

We investigated whether positive modulation of KCa3.1 by 1-ethyl-2-benzimidazolinone (1-EBIO) reversed the functional consequences of *CALM1* knockdown in HD T cells (Coleman et al., 2014). We conducted chemotaxis experiments in si*CALM1*-transfected HD T cells in presence or absence of 1-EBIO. Consistent with our earlier observation (**Figure 6E**), the Y-COM values of si*CALM1* transfected HD T cells were significantly reduced in the presence of adenosine (**Figure 7A**). However, when the si*CALM1* transfected T cells from the same individual were preincubated with 1-EBIO, the Y-COM values in the presence of adenosine were almost threefold greater than those of cells that were not preincubated with 1-EBIO (**Figure 7A**). Thus, addition of 1-EBIO blocked the inhibitory effect of adenosine in HD T cells transfected with si*CALM1* (**Figure 7B**). These data indicate that enhancing the channel function by using KCa3.1 activators could be effective in correcting the chemotactic defects of HNSCC T cells. A question remains whether KCa3.1 activators could be of additional benefit in augmenting the anti-tumor functionality of HNSCC T cells. We therefore tested the effect of 1-EBIO on INFγ production. We observed that 1-EBIO significantly enhanced IFNγ production in both scr-RNA as well as si*CALM1*-transfected HD T cells (**Figure 7C**). The positive effect of 1-EBIO on IFNγ production was observed both in wild-type HNSCC as well as HD T cells (**Figure 7D**). Overall, these data indicate that potentiating the KCa3.1 function has a positive and broad impact on the functional capabilities of CD8^+^ T cells of HNSCC patients.

## Discussion

The failure of immune surveillance in solid tumors has been ascribed, in part, to the inability of CD8^+^ T cells to infiltrate the tumor and function in the TME. Circulating cytotoxic T cells of HNSCC patients display a reduced ability to chemotax in an adenosine-rich tumor-like microenvironment; a limitation arising from a decrease in their KCa3.1 channel activity (Chimote et al., 2018). In the present study, we provide evidence of a compartmentalized reduction in CaM in HNSCC T cells that compromises the activity of KCa3.1 and the chemotactic ability of these cells.

CaM is a Ca^2+^ sensor ubiquitously expressed in cells and required for multiple functions as it binds many signaling and structural proteins (Feske et al., 2003; Hogan et al., 2003; Berchtold and Villalobo, 2014; Adelman, 2016; Anguita and Villalobo, 2018). Herein we showed that HNSCC circulating T cells express less CaM than HD T cells. The lower abundance of CaM in HNSCC T cells was due to the combination of a decrease in *CALM1* and *CALM2* and the failure of these cells to upregulate CaM upon activation. CaM levels have been shown to increase with activation in healthy T cells (Colomer et al., 1993). A decrease in *CALM1* has been reported for CD4^+^ TIL in breast cancer, and *CALM3* expression in TIL has been shown to be higher in extensively infiltrated tumors *vs* minimally infiltrated tumors suggesting a possible association between CaM levels and tumor infiltrating abilities of T cells (Gu-Trantien et al., 2013). Our data showed a plasma-membrane localized reduction of CaM in circulating T cells of HNSCC patients. There was no reduction in CaM in the rest of the cell. Indeed, we observed that the activity of KCa3.1 channels (a membrane-delimited CaM-dependent physiological process) of HNSCC T cells is affected by the reduction in membrane-proximal CaM, while other CaM-dependent functions like IFNγ production are still preserved.

KCa3.1 channels are Ca^2+^ dependent and require binding of intracellular Ca^2+^ to CaM, constitutively bound to the intracellular C-terminus of the channel, to open (Fanger et al., 1999; Khanna et al., 1999; Schumacher et al., 2001; Von Hahn et al., 2001; Stocker, 2004; Adelman, 2016; Lee and Mackinnon, 2018; Sforna et al., 2018). The importance of the CaM-KCa3.1 binding is underscored by the fact CaM co-immunoprecipitates with KCa3.1, and preventing the KCa3.1-CaM interaction by deletion of the C-terminus of KCa3.1, abrogates the channel function (Xia et al., 1998; Fanger et al., 1999; Khanna et al., 1999). Furthermore, addition of CaM antagonists prevents the binding of CaM to the channel and, consequently, reduces the KCa3.1 currents (Khanna et al., 1999; Von Hahn et al., 2001). This effect is reversed by addition of excess CaM intracellularly, suggesting that the KCa3.1-CaM interaction is reversible in nature (Khanna et al., 1999). While these studies have been conducted in heterologous expression systems, herein we are providing evidence that the KCa3.1-CaM interaction in CD8^+^ T cells is disrupted in pathological conditions like HNSCC. Reduced KCa3.1-CaM interaction ultimately results in reduced channel function. Our PLA data (**Figure 3**) showed a 57% reduction in the association of CaM with KCa3.1 in the cell membrane of HNSCC T cells, which was comparable to the 67% inhibition of KCa3.1 currents measured in HNSCC T cells (**Figure 4**). This decrease in KCa3.1 activity observed in HNSCC *vs.* HD T cells was comparable to the reduction in HNSCC T cells previously described by us (Chimote et al., 2018). Furthermore, we observed that delivery of CaM intracellularly through the patch pipette increased KCa3.1, but not Kv1.3 currents in HNSCC T cells. It has been recently reported that CaM binds to the beta subunit of Kv1.3 channels in transfected HEK-293 cells, however this binding does not alter the function of the Kv1.3 channels (Solé et al., 2019). Our data also indicate that increasing the free Ca^2+^ concentration in the pipette did not increase the KCa3.1 activity in HNSCC T cells, thus indicating that CaM deficiency, and not a reduction in Ca^2+^ sensitivity due to other mechanisms such as changes in CaM and/or channel phosphorylation, is responsible for low KCa3.1 activity in HNSCC T cells (Fanger et al., 1999). The Ca^2+^ sensitivity of the channel is also modulated by kinases and phosphatases, like protein kinase A (PKA) and nucleoside diphosphate kinase B (NDPK-B) (Gerlach et al., 2001; Srivastava et al., 2005; Srivastava et al., 2006a; Srivastava et al., 2006b; Srivastava et al., 2008; Srivastava et al., 2009; Di et al., 2010; Wong and Schlichter, 2014). Although we cannot exclude that alterations in these other pathways may exist in HNSCC T cells, our data clearly support a role for CaM reduction in HNSCC T cell dysfunction. In contrast to our findings in HNSCC T cells and to what was previously shown in other cell types, delivery of CaM intracellularly to HD T cells did not increase their KCa3.1 activity (Khanna et al., 1999; Joiner et al., 2001). It is possible that healthy T cell have sufficient amounts of intracellular CaM available to bind all the KCa3.1 channels on the membrane, which will leave the channel activity unchanged in presence of excess CaM. The contribution of CaM deficiency to the dysfunctional behavior of HNSCC T cells is further confirmed in HD T cells where knock-down of CaM conferred to these cells the same phenotype of HNSCC T cells. Silencing *CALM1* expression in HD T cells decreased KCa3.1 conductance (**Figure 6B**) to levels comparable to those of wild-type HNSCC T cells (**Figure 4B**) (Chimote et al., 2018). Furthermore, knock down of *CALM1* increased the sensitivity to adenosine of the HD T cells achieving an effect on chemotaxis comparable to that reported previously in HNSCC T cells (Chimote et al., 2018).

In accordance to what was previously shown in HNSCC T cells, our data showed that CaM deficiency reduces KCa3.1 activity, but not expression (Chimote et al., 2018). While it is well established that CaM regulates KCa3.1 activity, there are also reports that it facilitates the assembly of the pore-forming subunits and the surface trafficking of Ca^2+^-activated K channels (Joiner et al., 2001; Lee et al., 2003). However, despite the decrease in CaM abundance in HNSCC T cells or when we knocked down CaM in HD T cells, we did not observe a reduction in KCa3.1 surface expression (Chimote et al., 2018). This could be attributed to a difference in the cell type studied as well as the fact that CaM is not solely responsible for the trafficking of the channel to the plasma membrane; leucine zipper domains on the C-terminus of KCa3.1 have been implicated as well (Khanna et al., 1999; Syme et al., 2003). This raises the possibility that even though when CaM levels are decreased, alternate pathways may still be able to effectively traffic the KCa3.1 channels to the cell membrane.

IFNγ production is also Ca^2+^- and CaM-dependent. CaM regulates calcineurin activation, nuclear translocation of NFAT and production of downstream associated cytokines like IL-2 and IFNγ (Feske et al., 2003; Savignac et al., 2007; Liu, 2009). We did not observe a reduction in IFNγ production either in HNSCCC T cells or in *CALM1* transfected HD T cells. Instead, HNSCC T cells produced more IFNγ than HD T cells upon stimulation (**Figure 5A**). HNSCC and HD activated T cells display similar cytoplasmic CaM (**Figure 2C**), Ca^2+^ fluxing abilities (**Figure 5B**), and Kv1.3 channel activity (**Figure 4C**). The latter is sufficient to maintain the membrane hyperpolarization necessary for Ca^2+^ influx (Cahalan and Chandy, 2009). While these results can explain equal IFNγ production in HNSCC and HD T cells, the increase in IFNγ in HNSCC T cells may be due to enhanced TCR signaling upstream to ion channels. A robust increase in IFNγ in activated HNSCC lymphocytes (4-fold higher than in HD) was previously reported by others (Conti-Freitas et al., 2012).

Ultimately, herein we provide evidence that the negative functional consequences of CaM reduction in HNSCC T cells could be rescued by 1-EBIO. We have previously reported that activation of KCa3.1 channels by 1-EBIO or NS309 restored KCa3.1 function in HNSCC CD8^+^ T cells and abrogated the inhibitory effect of adenosine (Chimote et al., 2018). Eil et al. have recently shown that 1-EBIO increased INFγ production in mouse T cells (Eil et al., 2016). In this study, we showed that KCa3.1 activators can be used as a modality to correct the chemotactic abnormalities associated to CaM deficiency in CD8^+^ T cells of cancer patients. We also show that they can enhance INFγ production in both HNSCC and HD T cells indicating that this effect is independent of the CaM-deficiency and it is a consequence of the increased Ca^2+^ driving force that is achieved by activating KCa3.1 channels. This finding strengthens the therapeutic potentials of KCa3.1 activators, which could restore cytotoxic T cell functionality, and ultimately favor increased tumor infiltration and enhanced anti-tumor activity.

## Data Availability Statement

All datasets generated for this study are included in the article/**Supplementary Material**.

## Ethics Statement

The studies involving human participants were reviewed and approved by the University of Cincinnati Institutional Review Board. The patients/participants provided their written informed consent to participate in this study.

## Author Contributions

Conception and design: AC, and LC. Development of methodology: AC, and LC. Acquisition of data: AC, VG, and HN. Provision and management of patients: TW-D. Provision of patient data: TW-D. Analysis and interpretation of data: AC, VG, HN, TW-D, and LC. Writing, review, and/or revision of the manuscript: AC and LC. Study supervision: LC. All authors discussed the results and commented on the manuscript.

## Funding

This work was funded by grant support from the NIH (grant R01CA95286) to LC. HN received grant support from the NIH (T32CA117846T). TW-D was supported by a Clinical and Translational Science Award (CTSA)–awarded KL2 Mentored grant and a grant from CCTST (1UL1TR001425-01).

## Conflict of Interest

The authors declare that the research was conducted in the absence of any commercial or financial relationships that could be construed as a potential conflict of interest.

## Supplementary Material

Suplementary Table S1 Clinical and pathological characteristics of HNSCC patients.

Suplementary Table S2 CALM1 expression in HNSCC cells calculated with 18S rRNA and GAPDH as housekeeping genes.

Suplementary Table S3 Cell capacitance of activated T cells isolated from HD and HNSCC patients used in electrophysiological experiments.

Supplementary Figure 1 Assessment of background staining for KCa3.1-biotin antibody.

Supplementary Figure 2 Specificity of rabbit anti-human CaM antibody used for PLA experiments.

Supplementary Figure 3 Technical controls for PLA.

Supplementary Figure 4 KCa3.1 and CaM staining of HD and HNSCC T cells.

Supplementary Figure 5 KCa3.1 expression and activation status of HD T cells transfected with siCALM1.
