# Supplementary material for: A Compartmentalized Reduction in Membrane-Proximal Calmodulin Reduces the Immune Surveillance Capabilities of CD8+ T Cells in Head and Neck Cancer
Source: Front Pharmacol. 2020 Feb 28;11:143. doi: 10.3389/fphar.2020.00143 (PMC7059094; doi:10.3389/fphar.2020.00143)
Supplement: Suplementary Table S1 — Clinical and pathological characteristics of HNSCC patients. [file DataSheet_1.docx]

**Table S1. Clinical and pathological characteristics of HNSCC patients**. Clinical staging, tumor location and disease progression information for each HNSCC patient are shown. Time to median follow up was 13 months (Range 2-117 months). Pathological features include HPV status (p16), and grading. (N/A= data not available)

|  | Patient ID | Head and Neck Disease Site | Clinical Stage |  | p16 status | Differentiation | Disease Progression |
| --- | --- | --- | --- | --- | --- | --- | --- |
| 1 | HNC-70 | Oropharynx | T2N2bM0 |  | Positive | Poorly differentiated | No |
| 2 | HNC-72 | Oropharynx | T2N2bM0 |  | Positive | Poorly differentiated | No |
| 3 | HNC-73 | Oropharynx | T1N2bM0 |  | Positive | Poorly differentiated | No |
| 4 | HNC-74 | Oropharynx | T4aN0M0 |  | Positive | Moderately differentiated | No |
| 5 | HNC-79 | Oral Cavity | T2N2cM0 |  | N/A | Moderately differentiated | No |
| 6 | HNC-83 | Oropharynx | T1N2bM0 |  | Positive | Poorly differentiated | No |
| 7 | HNC-84 | Oropharynx | T1N2bM0 |  | Positive | N/A | No |
| 8 | HNC-120 | Larynx | T3, N0, M0 |  | Negative | Poorly to moderately differentiated | No |
| 9 | HNC-133 | Oropharynx | T4N2bM0 |  | Negative | Moderately differentiated | No |
| 10 | HNC-145 | Oral Cavity | T2N2bM0 |  | Positive | Poorly differentiated | No |
| 11 | HNC-148 | Oral Cavity | T1N1M0 |  | N/A | N/A | No |
| 12 | HNC-150 | Larynx | T4aN2bM0 |  | Negative | Poorly Differentiated | No |
| 13 | HNC-151 | Oral Cavity | T4aNxM0 |  | N/A | Well differentiated | No |
| 14 | HNC-152 | Oral Cavity | T2N0M0 |  | Negative | Well differentiated | No |
| 15 | HNC-155 | Larynx | T2N0M0 |  | N/A | Poorly to moderately differentiated | No |
| 16 | HNC-158 | Larynx | T3N0M0 |  | Negative | Moderately to well differentiated | No |
| 17 | HNC-160 | Hypopharynx | T2N3bM0 |  | Negative | Poorly differentiated | No |
| 18 | HNC-161 | N/A | T2N2cM0 |  | N/A | Poorly differentiated | No |
| 19 | HNC-166 | Oral Cavity | T3N0M0 |  | Negative | Moderately differentiated | No |

**Table S2. *CALM1* expression in HNSCC cells calculated with *18S rRNA* and *GAPDH* as housekeeping genes.** *CALM1* expression in resting and activated HNSCC T cells quantified by reverse transcription quantitative polymerase chain reaction (RT-qPCR). Data are the fold change in *CALM1* expression (calculated as RQ values, see Materials and Methods) relative to either *18S rRNA* expression or *GAPDH* expression. The data were normalized to mean resting *CALM1* expression. *GAPDH* expression was also measured in resting and activated HNSCC T cells relative to *18s rRNA* and normalized to resting *GAPDH* expression. Data are mean ± SEM from samples run in triplicate from four HNSCC patients. Data were analyzed by paired Student’s t test.

|  | **Resting** | **Activated** | **P-value** |
| --- | --- | --- | --- |
| *CALM1* vs *18s rRNA* | 1.053 ± 0.086 | 0.373 ± 0.037 | 0.008 |
| *CALM1* vs *GAPDH* | 1.047 ± 0.106 | 0.352 ± 0.073 | 0.003 |
| *GAPDH* vs *18s rRNA* | 1.043 ± 0.102 | 1.147 ± 0.161 | 0.309 |

**Table S3. Cell capacitance of activated T cells isolated from HD and HNSCC patients used in electrophysiologcal experiments**. Data are means ± SEM. ^a^p=0.0819 vs. HNSCC + CaM , ^b^p=0.182 vs. HD + CAM, ^c^p=0.673, ^d^p=0.141 vs. HD + siCALM1. Statistical significance in ^c^ determined by one way ANOVA, all other p-values measured by Student’s t-test.

|  | **Pipette Ca^2+^** | **Pipette CaM** | **Transfection** | **Capacitance (pF)** | **n** |
| --- | --- | --- | --- | --- | --- |
| HNSCC | 1 μM | - |  | 3.306 ± 0.136^a^ | 22 cells, 4 donors |
|  | 1 μM | 50 μM |  | 3.600 ± 0.436 | 22 cells, 4 donors |
| HD | 1 μM | - |  | 3.677 ± 0.149^b^ | 15 cells, 3 donors |
|  | 1 μM | 50 μM |  | 3.877 ± 0.111 | 15 cells, 3 donors |
| HNSCC | 1 μM | - |  | 3.613 ± 0.125^c^ | 18 cells, 3 donors |
|  | 3 μM | - |  | 3.812 ± 0.118 | 18 cells, 3 donors |
|  | 10 μM | - |  | 3.688 ± 0.180 | 18 cells, 3 donors |
| HD | 1 μM | - | Scr-RNA | 4.394 ± 0.354^d^ | 10 cells, 2 donors |
|  | 1 μM | - | siCALM1 | 3.677 ± 0.542 | 10 cells, 2 donors |


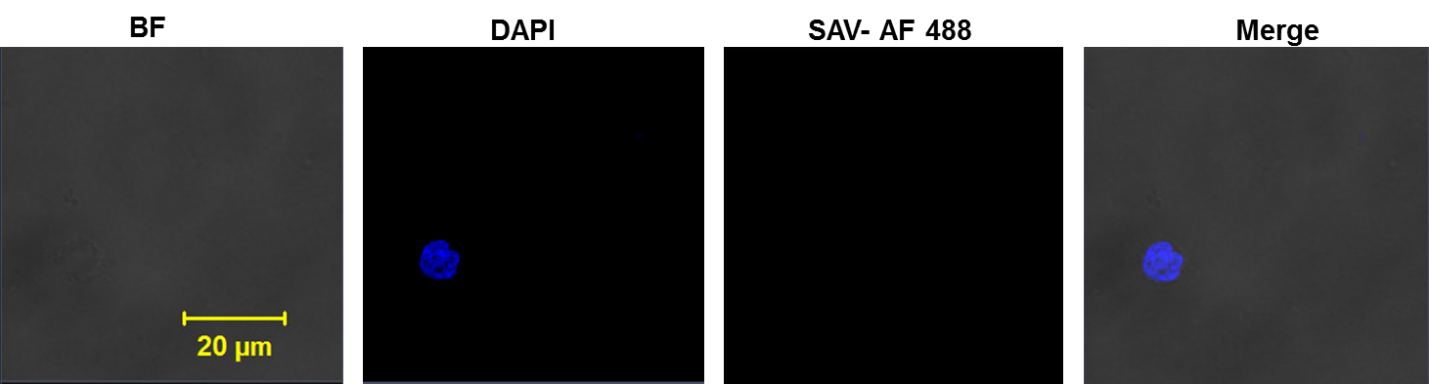


**Fig. S1.** **Assessment of background staining for KCa3.1-biotin antibody** Activated T cells from two healthy donors were stained with only the secondary antibody that was used for biotin conjugated KCa3.1 antibody used in the immunofluorescence experiments. Cells were plated on poly-l-lysine coated coverslips, fixed, blocked and stained with Alexa Fluor 488 conjugated streptavidin antibody (Biolegend) at a 1:50 fold dilution without addition of the primary antibody. Nuclei were stained with DAPI (blue) and images were acquired by confocal microscopy at 63× magnification as described in Materials and Methods. Shown here is a confocal image from a representative field from a single HD.

**
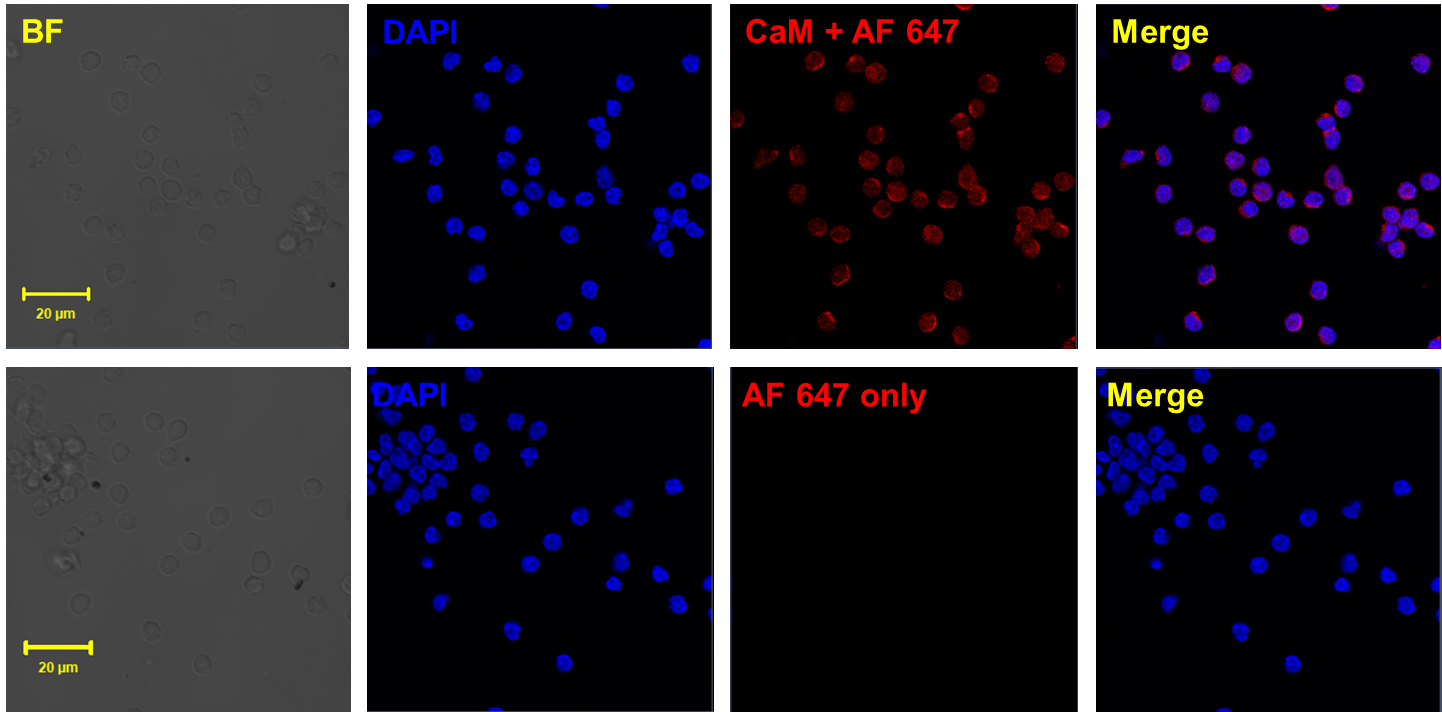
**

**Fig. S2.** **Specificity of rabbit anti-human CaM antibody used for PLA experiments** Activated T cells from a healthy donor were stained with monoclonal rabbit anti-human CaM antibody used in the immunofluorescence experiments. Cells were plated on poly-l-lysine coated coverslips, fixed, permeabilized and stained with monoclonal rabbit anti-human CaM antibody (Abcam). Cells were then stained with Alexa Fluor 647 conjugated donkey anti-rabbit antibody (ThermoFisher) at a 1:50 fold dilution (red). Cells stained with only the secondary antibody were used a controls. Nuclei were stained with DAPI (blue) and confocal images were acquired by confocal microscopy at 40× magnification. Shown here is a confocal image from a representative field from a single HD. (BF = brightfield)


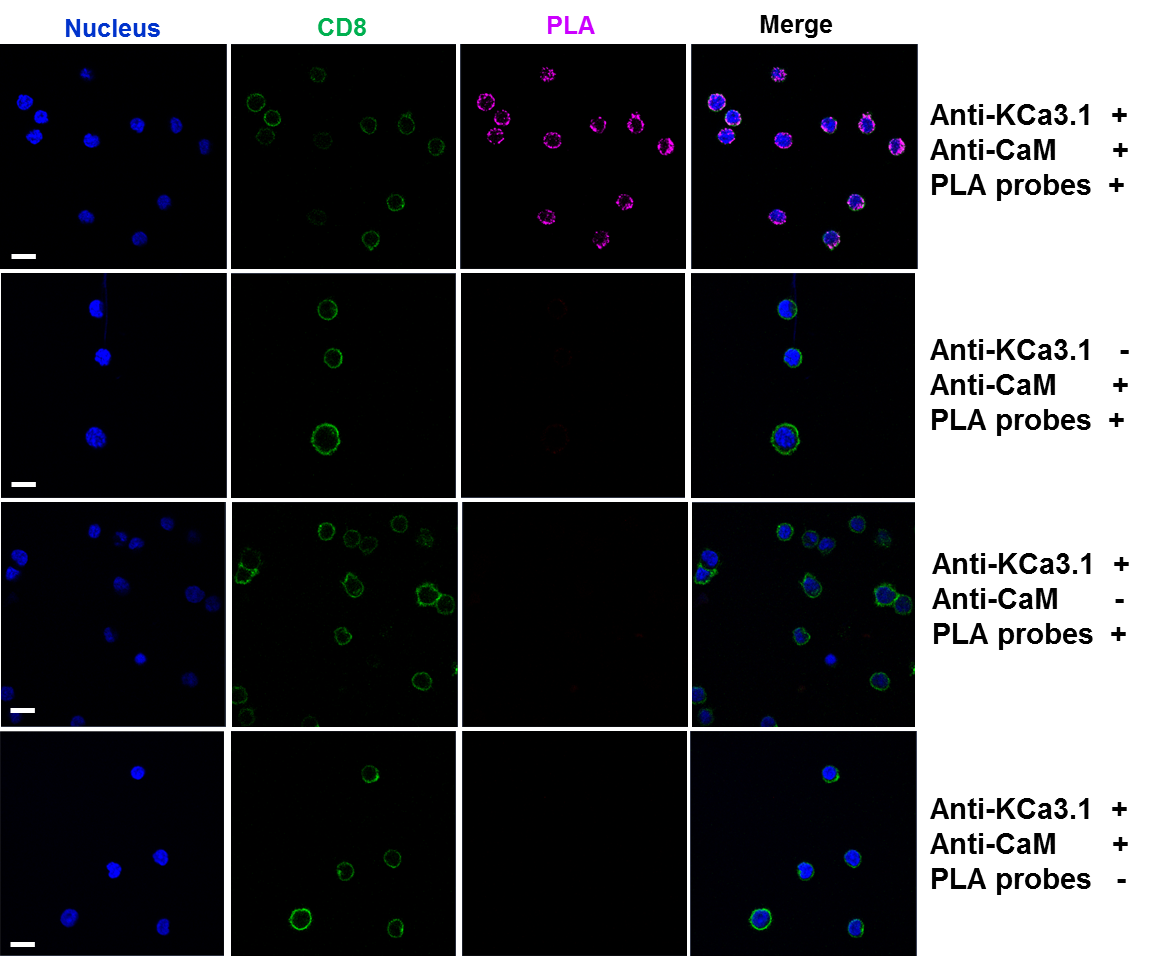


**Fig. S3.** **Technical controls for PLA** As technical controls for non-specific binding, PLA was performed by omitting either the KCa3.1 or CaM antibodies or PLA probes, as described in Materials and Methods. Shown here are representative confocal images acquired at 100X magnification for each condition. Top row, positive control: both primary antibodies and both PLA probes were added. Magenta dots represent positive PLA. As controls, PLA was performed by omitting either the anti- KCa3.1 primary antibody (second row), or anti-CaM primary antibody (third row), or the PLA probes (fourth row). Cell membrane was labeled with Alexa Fluor 647 conjugated anti-CD8 antibody (green) and nuclei were labeled with DAPI (blue). Scale bar, 10μm. The representative fields shown here are from a single HD. The technical control experiments were performed on two HDs and 2-3 fields were imaged per condition for each donor.

**
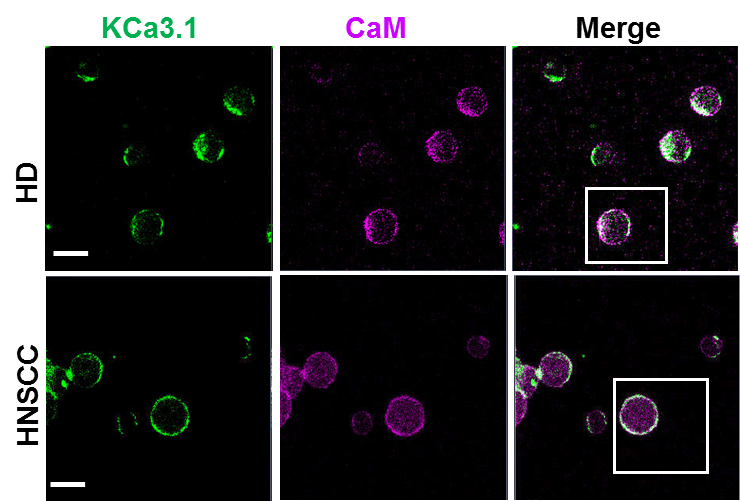
**

**Fig. S4.** **KCa3.1 and CaM staining of HD and HNSCC T cells** Representative confocal images of HD and HNSCC T cells stained for KCa3.1 (green) and CaM (magenta). Staining was performed in 3 HD and 3 HNSCC individuals, 5-10 fields were acquired for each donor. Merged images showing positive staining for KCa3.1 and CaM were used for line scan analysis (presented in Fig. 3). Representative magnified images of single HD and HNSCC T cells highlighted by the box in the “merge” channel are shown in fig. 3A. Scale bars indicate 10 μm.


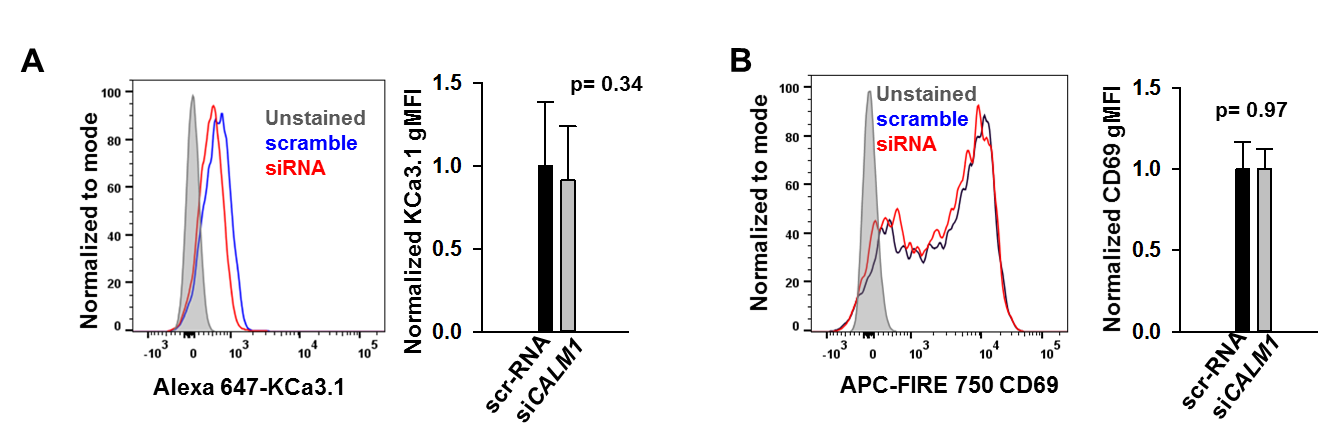


**Fig. S5.** **KCa3.1 expression and activation status of HD T cells transfected with si*CALM1* (**A) KCa3.1 and (B) CD69 expression (measured as gMFI) in T cells from three HDs transfected with either scr-RNA or si*CALM1*. Cells were gated on GFP-positive live cell population indicating successful transfection. Bars represent mean ±SEM of gMFI of either KCa3.1 (A) or CD69 (B) for each transfection condition. Data were analyzed by paired Student’s t-test.
